# Tissue morphology influences the temporal program of human brain organoid development

**DOI:** 10.1016/j.stem.2023.09.003

**Published:** 2023-10-05

**Authors:** Ilaria Chiaradia, Ivan Imaz-Rosshandler, Benedikt S. Nilges, Jerome Boulanger, Laura Pellegrini, Richa Das, Nachiket D. Kashikar, Madeline A. Lancaster

**Affiliations:** 1 MRC Laboratory of Molecular Biology, Cambridge Biomedical Campus, Cambridge, UK; 2 Resolve Biosciences GmbH, Alfred-Nobel-Strasse 10, 40789 Monheim am Rhein, Germany; 3 Wellcome-MRC Cambridge Stem Cell Institute, University of Cambridge, Cambridge, UK

**Keywords:** organoids, brain, development, stem cells, neurogenesis, morphology, spatial transcriptomics, cytoarchitecture, differentiation, cell fate

## Abstract

Progression through fate decisions determines cellular composition and tissue architecture, but how that same architecture may impact cell fate is less clear. We took advantage of organoids as a tractable model to interrogate this interaction of form and fate. Screening methodological variations revealed that common protocol adjustments impacted various aspects of morphology, from macrostructure to tissue architecture. We examined the impact of morphological perturbations on cell fate through integrated single nuclear RNA sequencing (snRNA-seq) and spatial transcriptomics. Regardless of the specific protocol, organoids with more complex morphology better mimicked *in vivo* human fetal brain development. Organoids with perturbed tissue architecture displayed aberrant temporal progression, with cells being intermingled in both space and time. Finally, encapsulation to impart a simplified morphology led to disrupted tissue cytoarchitecture and a similar abnormal maturational timing. These data demonstrate that cells of the developing brain require proper spatial coordinates to undergo correct temporal progression.

## Highlights

• Neural organoid protocol variations influence morphology and tissue architecture
• Advanced morphology correlates with greater transcriptomic similarity to *in vivo*
• Proper cellular position correlates with accurate fate progression
• Perturbing morphology and cytoarchitecture disrupts temporal identity

## Graphical abstract

**Figure:**
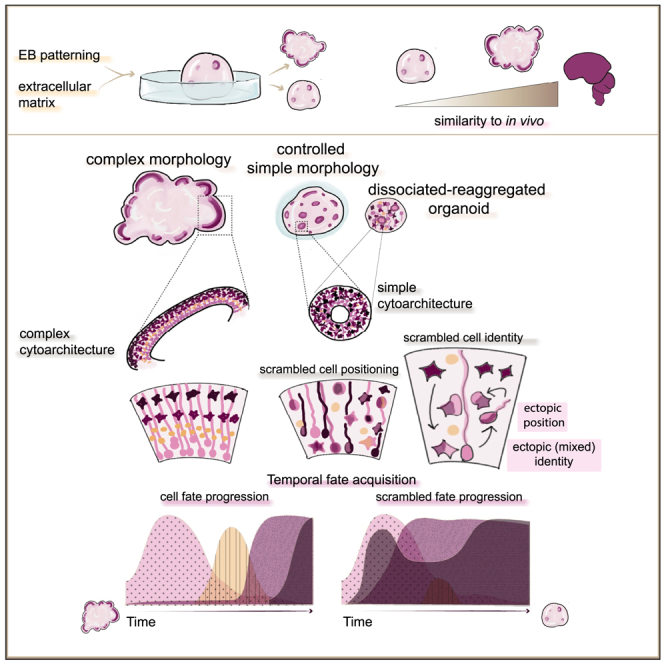

## Teaser

Proper cell fate acquisition in time and space determines tissue architecture, but how tissue architecture influences cell fate is less clear. Lancaster and colleagues investigated this form-fate relationship in brain organoids, revealing that tissue morphology and cell positioning in space influence the progression of cell identity in time.

## Introduction

Tissues rely on precise genetic information to establish their architecture and function. This causality has been widely studied in different tissue types, but relatively little is known about the opposite: how tissue organization may influence developmental programs over time.^1^^,^^2^ The nervous system is an exquisite example of collaboration between tissue structure and gene expression. The cortex develops with a defined architecture, forming large cortical hemispheres composed of discrete layers of progenitors and neurons along the apicobasal axis. However, the contribution of this tissue architecture to developmental fate progression, and whether such defined cellular positioning matters, remains to be examined.

*In vitro* models are powerful tools to examine the consequences of perturbing shape. For example, spatial confinement of embryonic stem cells was demonstrated to recapitulate germ-layer formation.^3^ More recently, scaffold-guided intestinal organoids displayed regional specificity in their cell type composition according to local curvature imparted by the scaffold.^4^ Likewise, brain organoids represent a valuable model to investigate tissue architecture and cellular identity determination, as they form complex morphologies and architecture through self-organization, just as *in vivo*. Such three-dimensional (3D) neural systems more closely resemble the *in vivo* developmental transcriptome^5^^,^^6^ and display a greater complexity of cell types^7^ compared with more simple two-dimensional (2D) rosettes, suggesting that tissue structure may influence proper acquisition of cell fate.

Numerous recent studies have analyzed the transcriptomic profile of neural organoids in comparison with *in vivo*, which have revealed sometimes contradictory issues with the fidelity of organoids as a model.^8^^,^^9^^,^^10^^,^^11^ However, little attention has been paid to organoid morphology as a potential variable in helping explain differences in expression profiles. Many methods for neural organoids now exist, but despite the similarity in brain regional identity and gross cell composition^12^^,^^13^ of different protocols, the overall morphology of the tissue can be markedly different from one protocol to the other. For instance, human cortical spheroids form small, homogeneous spherical aggregates with numerous neural rosette-like structures,^14^ whereas undirected brain organoids form convoluted neuroepithelia and large lobes with fluid-filled ventricles.^15^^,^^16^ The source of this disparity in organoid morphology, and whether these differences influence identity determination, is yet to be answered.

Here, we investigated the interdependency of tissue structure and identity using brain organoids. By systematically testing key protocol variations, we identify factors shaping organoid morphology. We find that gross morphology can predict tissue architecture, and manipulating morphology results in changes to organoid architecture. We then examine how the spectrum of morphological diversity influences cell fate through single-nucleus and single-cell transcriptomics. This reveals a link between tissue shape and tissue fate with a common signature shared among independent morphological perturbations, with organoids displaying a more complex morphology having greater transcriptional similarity to the *in vivo* counterpart. Moreover, manipulating cell position to create a scrambled cytoarchitecture or imparting a simplified morphology and cytoarchitecture results in aberrant temporal fate progression, such that when cells lose their position in space, they also lose their maturational timing.

## Results

### Organoid patterning is a key regulator of tissue morphology

**Figure 1:**
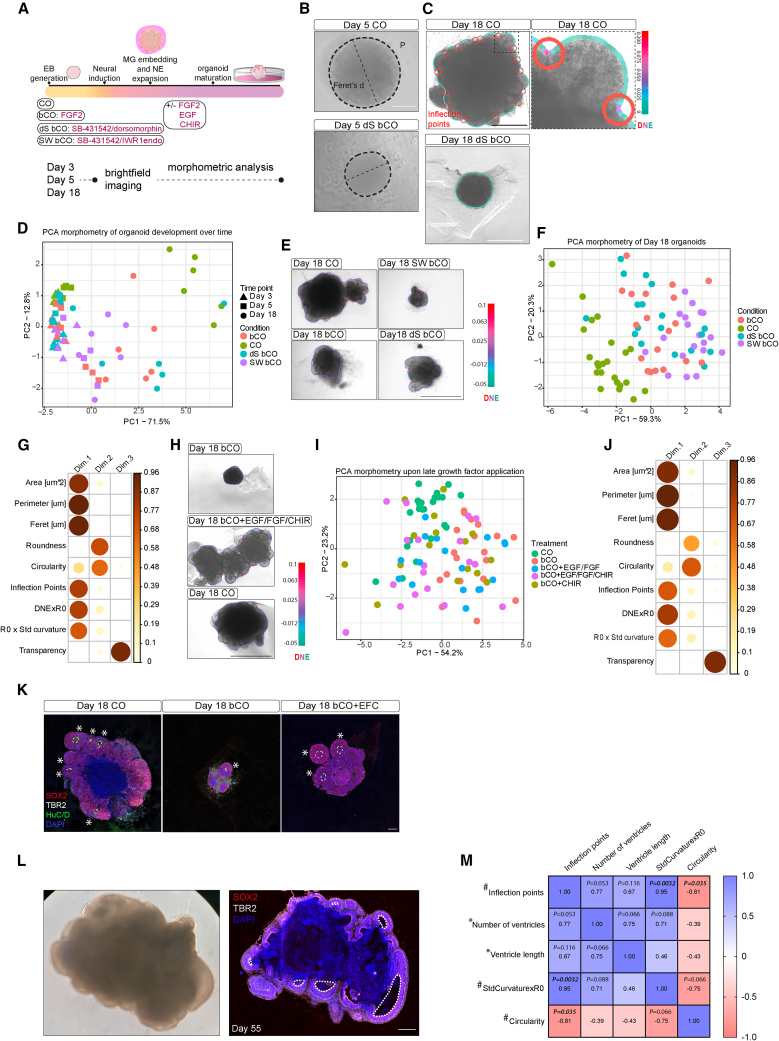
Common protocol variations affect organoid morphology (A) Schematic of the protocol and the variables tested. Guided (dS bCO, SW bCO) and unguided or minimally guided protocols (bCO, CO), Matrigel (MG) embedding, and neuroepithelial expansion (+/− FGF2 [bFGF], EGF, and CHIR). Organoids were imaged on days 3, 5, and 18 and processed for morphometric analysis. (B) Representative bright-field images of day 5, showing perimeter (P) (dashed contour) and Feret’s diameter (dashed diameter). (C) Representative images of day 18 organoids showing DNE (contour line) and inflection points (red circles) on CO and dS bCO. Inset shows a higher-magnification image of one lobule. Scale bar indicates DNE (Dirichlet normal energy). (D) Principal-component analysis (PCA) of morphological measurements revealing the morphospace of organoid development over time. Principle component (PC) axes display the percentage of variation. Data points represent the batch average of n = 3–10 organoids. At day 3, six batches per condition were analyzed with the exception of CO (five batches). At day 5, six batches per condition were analyzed, with the exception of dS bCO and CO (five batches each). At day 18, six batches per condition were analyzed, with the exception of dS bCO and CO (five batches). (E) Representative bright-field images at day 18 processed through morphometric analysis. Gradient scale indicates DNE and red circles indicate inflection points. (F) Morphospace of individual day 18 organoids from six batches per condition, with the exception of dS bCO and CO (five batches). PC axes display the percentage of variation. (G) Correlation plot of the PCA analysis shown in (F) showing the contribution of each morphometric parameter as the squared cosine across principal components. Color intensity and size of the circles are proportional to the contribution of each parameter to the principal components. (H) Representative bright-field images of day 18 growth factor treated and untreated organoids processed through the morphometric analysis. Gradient scale indicates DNE, and red circles indicate inflection points. (I) Morphospace of day 18 treated and untreated single organoids from six batches. PC axes display the percentage of variation. (J) Correlation plot of the PCA shown in (I). (K) Immunohistochemistry of day 18 unguided CO, bCO, and bCO treated with EGF/FGF/CHIR showing progenitors (SOX2+), IPCs (TBR2+), neurons (HuC/D+), and nuclei (DAPI+). Neural tube-like lobules are shown by asterisks, and ventricles are outlined by a dotted line. (L) Bright-field and immunohistochemistry for radial glia (SOX2+), IPCs (TBR2+), and nuclei (DAPI+) of the same organoid at day 55. Dotted lines outline the ventricles. (M) Spearman correlation of parameters measured through the morphometry pipeline (denoted by #) and macroarchitecture parameters measured by immunohistochemistry (denoted by ^∗^). 7 organoids from 1 batch were analyzed. For correlation values above |0.5|, p values are shown, and they are in bold for p < 0.05. Two-tailed nonparametric Spearman correlation was performed. Scale bars: 200 μm in (B), 500 μm in (C), 1,000 μm in (E) and (H), 100 μm in (K), and 500 μm in (L).

We first examined how different variations in cerebral organoid protocols could influence tissue morphology. Systematic comparison of published protocols revealed key variables that consistently differed among protocols (Table S1). We tested embryoid body (EB) patterning with small molecules, Matrigel (MG) exposure, and growth factors during neuroepithelial expansion while maintaining other variables constant. This resulted in unguided cerebral organoids in basal medium (following Lancaster et al. protocol^15^) (bCO), cerebral organoids made according to an optimized protocol^17^^,^^18^ with a proprietary media but still lacking patterning molecules (CO), and guided cerebral organoids treated with dualSMAD (transforming growth factor β [TGF-β] and bone morphogenetic protein [BMP]) inhibitors (dS bCO) or SMAD WNT (TGF-β and WNT) inhibitors (SW bCO) (Table S2; Figure 1A; Figure S1A). We developed an unbiased semi-automated pipeline measuring nine morphological parameters including size, geometry, texture, and surface complexity descriptors (see STAR Methods; Figure S1B) at days 3, 5, and 18 to capture the effects on early EB morphology and later neuroepithelial morphology (Figures 1A–1C).

Previous studies have demonstrated unbiased multivariate image analysis to uncover how perturbations influence morphology.^19^ We similarly applied principal-component analysis (PCA) to the measurements of organoid morphology. Datapoints corresponding to averaged organoid profiles within each independent batch and across all time points were plotted in morphospace.^20^ This revealed a higher distribution area of day 18 organoids compared with embryoid bodies (Figure 1D), particularly along principal component 1 (PC1) or dimension 1 (dim.1), represented by size and surface complexity descriptors (Figure S1C), indicating an increase in morphological complexity as organoids matured. COs displayed a consistent morphological distance from the other conditions regardless of the time point, but the difference became greater at day 18 with a more complex surface pattern (Figure 1C). Bright-field images of COs compared with bCOs, dS bCOs, and SW bCOs at day 18 and PCA revealed that COs resided in a separate cluster, whereas guided (dS bCO, SW bCO) and bCO samples formed intermingled clusters (Figures 1E and 1F). Immunohistochemistry revealed that the difference in size and surface complexity (Figure 1G) was reflected by the difference in number of ventricles and ventricle size (Figure S1D).

Next, we tested the effect of commonly used growth factors that promote neural stem cell proliferation and maintenance,^21^ an approach also used in several organoid protocols.^14^^,^^22^ Treatment with the combination of three factors (epidermal growth factor [EGF]/fibroblast growth factor [FGF]/GSK3 Inhibitor/WNT Activator [CHIR-99021]) shifted the morphospace of SW bCO, dS bCO, and bCO to different extents toward CO (Figure 1H and 1I; Figures S1E–S1J). The change affected organoid size (Figure 1J; Figures S1G and S1J) and the number of ventricles (Figure 1H; Figures S1E and S1H). All the conditions displayed a high degree of sample-to-sample variability in shape, but guided dS bCOs showed higher reproducibility in tissue shape compared with unguided bCOs (Figure S1K), which ranged from complex optimal morphology (Figure 1E) to suboptimal morphology (Figure 1H), consistent with reported literature.^9^

### Tissue morphology and architecture are related

Differences in gross morphology could reflect changes in tissue structure and composition. We examined macroarchitecture by immunohistochemistry and observed more and larger ventricles for growth factor-treated organoids compared with untreated (Figure 1K, asterisks). Quantification of TBR2+ (EOMES) intermediate progenitor cells (IPCs) and HuC/D+ neurons revealed a trend of decreased numbers of these more differentiated cell types in growth factor-treated organoids compared with untreated (Figure S1L), regardless of the starting condition.

Next, we performed Spearman correlation between parameters of surface complexity measured through semi-automated morphometry of bright-field images and macroarchitecture parameters measured by immunohistochemistry on the same organoids (Figure 1L). Morphological parameters of surface complexity (inflection points, circularity, and StdCurvature × R0) were highly correlated to each other, suggesting a synergy in describing organoid shape (Figure 1M). Inflection points positively correlated with the number of ventricles and with ventricle length, suggesting that organoids with more ventricles also have larger ventricles, which is supported by the positive correlation between ventricle number and ventricle length.

**Figure 2:**
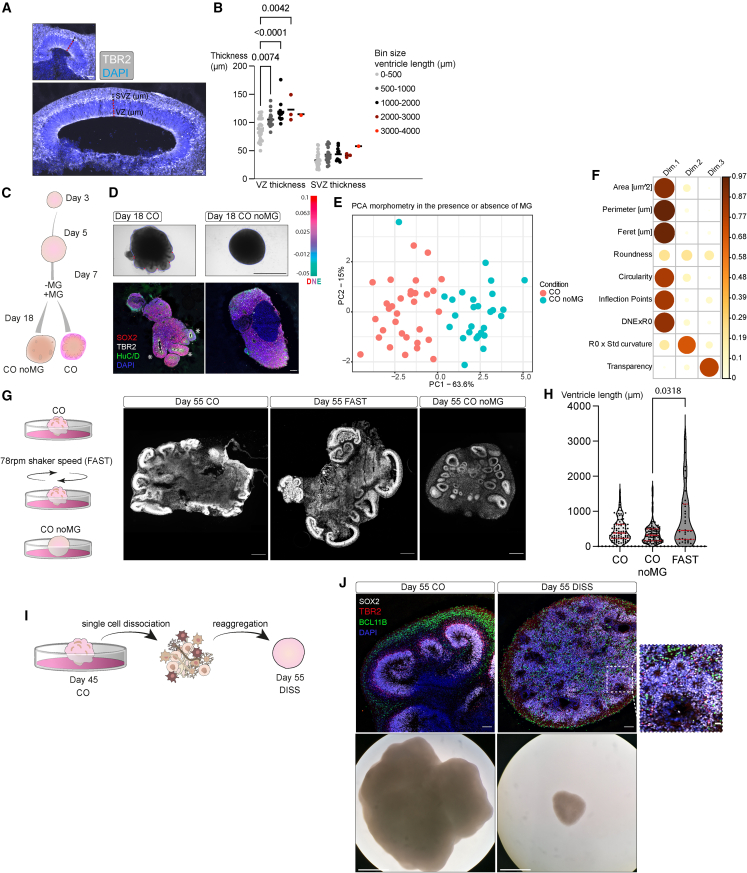
Morphology reflects tissue architecture (A) Representative images of a large and small ventricle and the respective VZ thickness (red dotted line) and SVZ thickness (black dotted line) from sectioned day 55 organoid stained for IPCs (TBR2+) and nuclei (DAPI+). (B) Quantification of VZ and SVZ thickness of ventricles grouped by ventricle length and displayed in bins of 0–500, 500–1,000, 1,000–2,000, 2,000–3,000, and 3,000–4,000 μm. Two-way ANOVA test was performed. Mean is shown in the graph. Only p < 0.05 from Tukey’s multiple comparisons test are shown. Data points represent individual ventricles. 17 organoids from two batches were analyzed. (C) Cartoon of CO and CO noMG conditions. The two conditions were treated identically and within the same batch until day 7, when MG was applied (CO) or excluded (CO noMG). (D) Top: representative bright-field images of CO (+MG) and CO noMG day 18 processed through the morphometric analysis. Gradient scale indicates DNE and red circles indicate inflection points. Bottom: immunohistochemistry showing progenitors (SOX2+), IPCs (TBR2+), neurons (HuC/D+), and nuclei (DAPI+). Asterisks mark lobules, and ventricles are outlined by a dotted line. No ventricles are visible in CO noMG at this early time point. (E) PCA of morphological measurements of day 18 CO (+MG), CO noMG. Principal component (PC) axes display the percentage of variation. Data points represent single organoids from six batches per condition. (F) Correlation plot of the PCA in (E) showing the contribution of each morphometric parameter as the squared cosine of each across principal components. Color intensity and size of the circles are proportional to the contribution of each parameter. (G) Cartoon of the conditions tested for affecting later organoid morphology and representative images of organoid macroarchitecture at day 55. Sections were stained for nuclei (DAPI+). (H) Violin plot of ventricle length in day 55 CO, FAST, and CO noMG. Data points represent individual ventricles. CO = 5 batches, CO noMG = 3 batches, and FAST = 3 batches. There were 1–5 organoids analyzed per batch. Median and quartiles are shown by dotted lines. One-way ANOVA test and Kruskal-Wallis test for multiple comparisons were performed. Only p < 0.05 from Kruskal-Wallis multiple comparisons test is shown. (I) Schematic of single-cell dissociation of day 45 CO, followed by reaggregation and analysis 10 days later (day 55 DISS). (J) Bright-field and immunohistochemistry images of day 55 CO and DISS labeled for VZ progenitors (SOX2+), SVZ progenitors (TBR2+), neurons (BCL11B+), and nuclei (DAPI+). The inset from DISS organoid shows the region with the highest degree of organization with small neural rosette-like structures. Scale bars: 50 μm in (A), 100 μm immunohistochemistry and 1,000 μm bright field in (D), 500 μm in (G), 100 μm immunohistochemistry, inset 20 μm, and bright field 1,000 μm in (J).

Lumen size has been shown to relate to cell shape and cell fate.^23^ We therefore examined cytoarchitecture—ventricular zone (VZ) and subventricular zone (SVZ) thickness—of small and large ventricles (Figure 2A). Larger ventricles exhibited increased VZ thickness with a similar pattern for SVZ thickness, though not significant (Figure 2B). Thus, organoids with higher surface complexity also displayed more and larger ventricles with increased proliferative layer thickness.

We next tested the effects of other perturbations that would impact morphology more directly, rather than through patterning. The use of MG varies across protocols, in some cases being used for embedding,^15^^,^^24^ added to the medium,^25^ or completely omitted.^14^ We tested the complete omission of MG from batches with otherwise identical media composition and protocol (Figure 2C) (Table S2). Organoids with no MG (CO noMG) varied significantly from the CO counterpart at day 18 and lacked large ventricles forming instead compact unpolarized tissues (Figure 2D—asterisks marking ventricles, dotted line marking ventricle size). The difference in morphology could be explained almost entirely by PC1 of the morphospace (Figure 2E). Among the variables contributing to PC1, size and surface complexity—circularity, inflection points, and Dirichlet normal energy (DNE) × R0—once again played a leading role in determining the morphology (Figure 2F).

Orbital shaker speed can vary across published protocols and ranges from 57,^26^ 80,^27^ to 90 rpm,^10^ and this speed would result in differences in centrifugal force and sheer force experienced by the organoids.^17^ We tested the effect of increasing the orbital shaker speed from 57 to 78 rpm (FAST condition), using otherwise identical media and protocol (Figure 2G; Table S2). We observed an overall wider distribution in ventricle length for FAST organoids compared with COs and noMG condition. Organoids never exposed to MG showed consistently smaller ventricles (Figures 2G and 2H). These results suggest that mechanical cues that influence gross morphology also influence macroarchitecture.

We next tested whether the opposite is true: whether perturbation of cytoarchitecture would influence overall morphology. To perturb cell position, we dissociated mature COs at day 45 and reaggregated the single-cell suspension (DISS), followed by analysis 10 days later to capture the signature of cytoarchitecture disruption over the stress signature that might arise following mechanical dissociation (Figure 2I). This led to a broad rearrangement of cytoarchitecture but overall maintained cell populations. DISS organoids completely lacked discernible ventricles, and the polarized SOX2+ VZ epithelium was lost, as was the spatial segregation into TBR2+ SVZ and BCL11B+ cortical plate (CP) (Figure 2J). In some regions, DISS organoids displayed very small rosette-resembling structures (Figure 2J inset), lacking any layering characteristic of ventricles. The morphology of DISS organoids was also simple and spherical, with no protruding ventricles (Figure 2J), demonstrating that cellular positioning could be effectively disrupted using this approach, leading to simplified cytoarchitecture and gross morphology.

### Tissue morphology and cell identity are related

**Figure 3:**
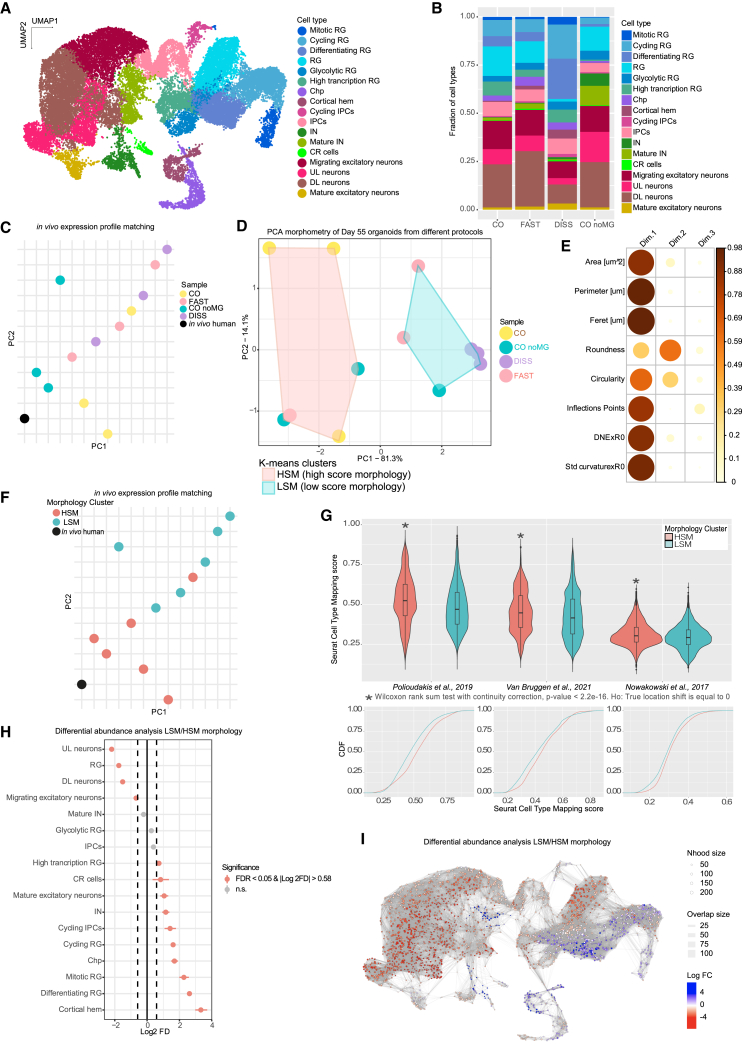
Morphology predicts organoid quality and transcriptional similarity to *in vivo* (A) Uniform manifold approximation and projection (UMAP) of the transcriptomic landscape from snRNA-seq of day 55 CO, CO noMG, FAST, and DISS organoids. Cells are colored by clusters. Three batches with one organoid each per condition were sequenced, except for CO noMG condition with three organoids from two batches. (B) Bar plot of cluster representation in the different conditions. (C) PCA plot generated from the average of scTransformed gene expression values across all cells of the above snRNA-seq samples compared with *in vivo* human developing brain scRNA-seq dataset.^28^ (D) PCA and k-means clustering of the top two PCs obtained from morphological measurements of the same day 55 organoids sequenced through snRNA-seq. Two clusters are highlighted, one containing organoids with complex morphology (HSM) and one with organoids with simplified morphology (LSM). (E) Correlation plot of the PCA shown in (D) showing the contribution of each morphometric parameter as the squared cosine across principal components. Color intensity and size of the circles are proportional to the contribution of every parameter to the principal component. (F) The same PCA plot as in (C). This time color-coded by morphology score (HSM, LSM). (G) Violin plots (top) showing comparison of Seurat cell type mapping scores between HSM and LSM cluster organoids, across multiple *in vivo* publicly available datasets. Statistically significant differences between the distributions of these two clusters are indicated by a Wilcoxon rank sum test. Cumulative distribution functions (CDFs) show that HSM groups are shifted toward larger values of cell type mapping scores (bottom). Ho (null hypothesis) is no shift in the distribution. (H) Permutation-based (scProportionTest) cell type composition analysis of LSM versus HSM samples. Significantly enriched populations are highlighted in red. FD, fold difference; FDR, false discovery rate. (I) MiloR cluster agnostic differential abundance analysis of LSM organoids versus HSM. The UMAP plot shows cell neighborhood connections using a shared nearest neighbor graph, highlighting over-represented (in blue) and under-represented (in red) regions in the transcriptomic landscape of LSM organoids. Nodes are neighborhoods (Nhood), colored by their log-fold change across conditions. Non-differentially abundant (DA) neighborhoods (FDR 5%) are colored in white, and sizes correspond to the number of cells in a neighborhood. Graph edges depict the number of cells shared between adjacent neighborhoods.

Whether morphology and tissue architecture influence the identity of the cells within is unclear. We examined the transcriptomic signature of morphologically distinct organoids. Since patterning molecules early on would directly influence cell fate and confound the interpretation of the influence of morphology, we selected the more direct morphological perturbations, namely CO noMG, FAST, and DISS, to compare with control CO, for further study. We performed single nuclear RNA sequencing (snRNA-seq) on day 55 organoids, resulting in 30,366 profiled cells from 12 independent organoids of 3 different batches. Clustering and differential gene expression analysis revealed the presence of distinct and variegate progenitor and neuronal subpopulations with cell type specific markers (Figure 3A; Figure S2A), similar to the cell types previously identified in cerebral organoids.^29^

We carried out cell type annotation based not only on cellular identity but also cell state/function. The radial glial (RG) progenitor cluster (*GLI3*+) was further subclustered based on cell cycle phase (Figures S2B and S2C; Table S3), metabolic status, and differentiation potential (Figures S2D–S2F). We succeeded in detecting abundant non-apical IPC and outer RG progenitors, the latter of which were detected within the RG cluster (Figures S2G and S2H). We also detected regions adjacent to the cortex—cortical hem and choroid plexus (Figures S2I and S2J). Several neuronal subtypes were detected—Cajal-Retzius (CR) cells, migrating neurons, interneurons (INs), deep-layer (DL) neurons, upper-layer (UL) neurons, and mature excitatory neurons (Figure 3A; Figures S2K–S2P). Intriguingly, one cluster lacked specific cell type identity and displayed high expression of genes associated with metabolic functions and proteostasis, including a large number of ribosomal genes, many of which were previously described to be in stressed cells of organoids.^30^ However, quality control (QC) post-analysis showed that these cells belonged with high probability to droplets with elevated contamination of ambient RNA (Figure S3). Hence, these pseudo-cells were removed from downstream analysis, resulting in 29,216 cells that passed QC (see STAR Methods for details).

Differential abundance analysis revealed a more striking phenotype in DISS samples (Figure 3B). Even though dissociated-reaggregated organoids came from the same cell line, culture condition, batch, and even the same dish as COs, the cytoarchitectural disruption significantly altered their cellular makeup. Differential abundance analysis performed with two independent methods—Milo-test^31^ and scProportionTest^32^—showed consistent differences between cell populations of DISS and CO conditions (Figures S4A and S4B). DISS organoids exhibited decreased UL, DL, and migratory neurons but an enrichment in other cell types such as cortical hem, differentiating RG, CR cells, and INs. This could reflect a differential effect of mechanical dissociation on certain cell types. Neurons are thought to be extremely sensitive to dissociation protocols. Interestingly, the dissociation and reaggregation resulted in the increase of cycling progenitors and differentiating RG (Figures S4A and S4B), suggesting a plastic response of the tissue to potential cell loss and cell stress.

No statistically significant differences were observed between FAST and CO in the annotation-independent Milo-test (Figure S4A). However, the scProportionTest highlighted the over-representation of cortical hem and CR cells in FAST organoids (Figure S4B), consistent with the observation from DISS organoids, pointing to potential similar differences, albeit milder in the case of FAST organoids. Comparison of CO noMG to CO revealed more striking differences. Alongside a consistent loss in certain RG cell types—high transcription RG, differentiating RG, and cycling RG—we also observed an increase in INs (Figure 3B; Figures S4A and S4B). These changes point to key differences in fate determination in the absence of extracellular matrix, which provides a scaffold but may also function in signaling in this context.^33^

A primary readout of organoid quality is its potential to mimic the developing brain both histologically and transcriptionally. We thus investigated how the different organoids sequenced matched the *in vivo* transcriptome. We compared our snRNA-seq data with publicly available data of human fetal brain^28^ representing a mid-gestation developmental stage (gestational week [GW] 17–18), which previous studies have demonstrated brain organoids are capable of recapitulating.^7^^,^^12^ To integrate with organoid data, the single-cell transcriptomic profile of each sample was collapsed to its mean gene expression and compared with one another through PCA (Figure 3C). We also calculated Seurat cell type mapping scores for each organoid sample compared with the *in vivo* dataset, which revealed that samples more distant from *in vivo* on the PCA plot generally exhibited lower mapping scores (Figure S4C).

The comparison to *in vivo* revealed some intriguing discrepancies within treatments. In general, the expression profile vicinity to *in vivo* could not be explained according to the treatment/protocol applied. Within a given treatment, single organoids showed variability in the transcriptional profile, particularly evident for CO noMG (Figures S4D and S4E). Before preparing snRNA-seq libraries on these organoids, we documented each one with bright-field images. We therefore examined the images from these noMG samples and observed a striking difference in their morphology, namely lobular structure and size (Figure S4D). Accordingly, CO noMG with more complex morphology showed better representation of the different cell types (Figure S4D) as well as closer similarity to *in vivo* (Figure S4E). These findings suggest that identity may relate more to the morphology of the organoid rather than the particular protocol applied.

### Scoring for morphology predicts organoid quality and similarity to *in vivo*

To test whether morphology may be a predictor of transcriptomic signature, we scored morphology of organoids processed for snRNA-seq using our morphometric analysis pipeline, focusing on size, geometry, and surface complexity descriptors (Figure S4F). PCA and unbiased k-means clustering resulted in two morphology clusters (Figure 3D). The separation between the two can be explained mainly by organoid size, DNE × R0, StdCurvature × R0, and inflection points from PC1 (Figure 3E). We labeled the clusters as high-score morphology (HSM) and low-score morphology (LSM) according to the degree of morphological complexity and the observation that the LSM cluster contained all the samples from the DISS condition, which were smaller and simplified.

Reanalysis of the PCA revealed that most HSM samples positioned close to the *in vivo* datapoint, compared with LSM (Figure 3F) suggesting that organoids with more complex morphology better mimicked the *in vivo* expression profile. To further test this statistically, we incorporated two more publicly available datasets of *in vivo* brain development.^34^^,^^35^ These two datasets were integrated following the same procedure with the calculation of cell type mapping score and a Wilcoxon sum rank test to avoid parametric assumptions. For all three datasets, HSM performed better in matching to *in vivo*, indicated by a significant shift in the mapping score distributions toward larger values (Figure 3G). This result indicates that morphology is correlated with transcriptome similarity to *in vivo* and thus could be used to predict organoid quality.

To investigate how the two morphology groups differed transcriptionally, we performed differential abundance analysis on the snRNA-seq landscape for LSM organoids versus HSM organoids. scProportionTest revealed a reduction in RG and excitatory neuron populations, with the strongest effect on UL neurons, while there was an increase in high transcription RG, differentiating RG, and cycling RG, as well as INs and cortical hem (Figure 3H). Milo-test showed a global rearrangement of cell type representation (Figure 3I) with a reduction of excitatory neurons and RG, while there was an enrichment in other RG populations and adjacent regions such as the cortical hem. LSM-enriched clusters exhibited upregulation of morphogenesis GO terms (Table S4), suggesting a morphogenetic response. These data suggest that morphology and tissue cell type composition are intimately linked.

### Spatial transcriptomics reveals discrete cell populations and cell positioning

Spatial transcriptomics offers unprecedented advantages of retaining both cell type and cell location coordinates.^36^ We performed Molecular Cartography *in situ* hybridization with one hundred transcripts based on known markers as well as transcripts we identified in the snRNA-seq, focusing on CO as a consistent example of HSM and on CO noMG as a condition offering a spectrum of different morphologies. The chosen gene set allowed us to identify the structural composition across different areas of the organoid (Figure S5A), with the vast majority of the genes classified as spatially variable markers according to Moran’s coefficient of spatial autocorrelation. The organoid architecture was well captured through spatially explicit dimensionality reduction (Figure S5B) (Table S5).

**Figure 4:**
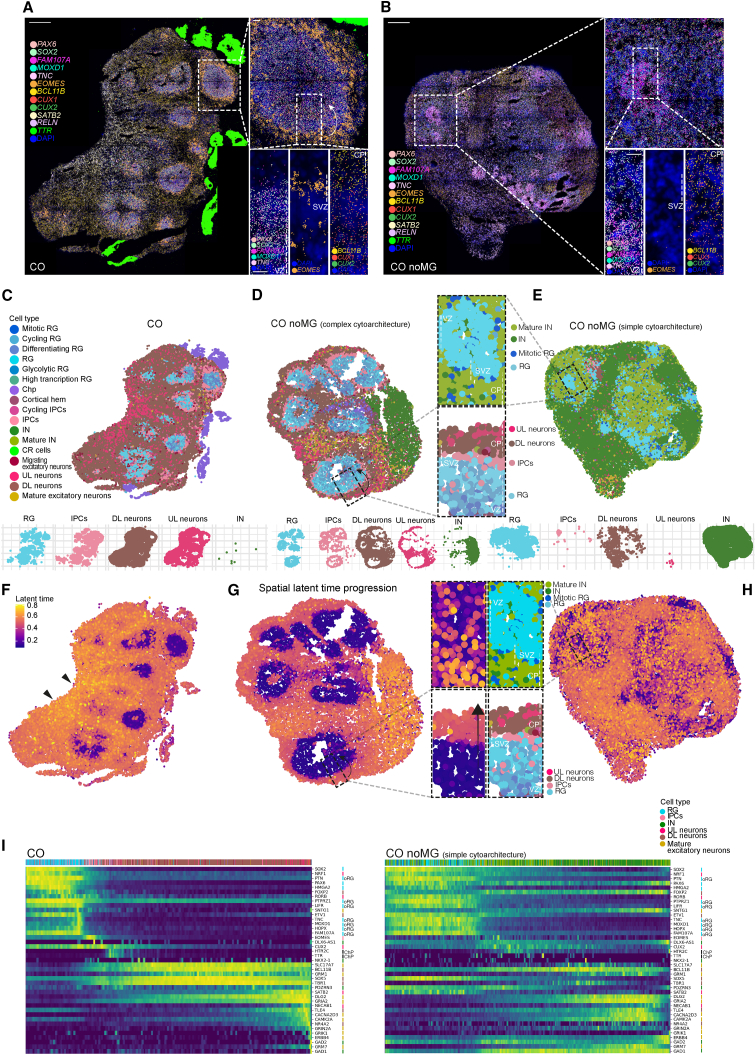
Spatial positioning of cells impacts temporal fate progression (A and B) Molecular Cartography of CO (A) and CO noMG with simple morphology (B). The transcripts shown at single-cell resolution are color coded. Zoomed-in view of a ventricle of CO and a rosette-like structure of CO noMG shows progenitors and neuron localization. Markers for RG (*PAX6*, *SOX2*, *FAM107A*, *MOXD1*, and *TNC*) localize in the VZ of CO but are interspersed in CO noMG. *EOMES*+ (*TBR2*) IPCs localize in the SVZ of CO, whereas sparse signal was detected in CO noMG with a simpler cytoarchitecture. *BCL11B*, *CUX1*, and *CUX2* are used as markers of DL and UL neurons, respectively. *RELN* marks CR cells, whereas *TTR* marks ChP. CP, cortical plate. (C–E) Superimposed snRNA-seq clusters onto the spatial map for CO (C), CO noMG with complex cytoarchitecture (D), and CO noMG with simple cytoarchitecture (E). The spatial map is the result of two averaged and superimposed technical replicates for CO, one for CO noMG with complex cytoarchitecture, and two biological replicates for CO noMG with simple cytoarchitecture. Spatial distribution of cell types by cluster for a selection of clusters (lower) (full cluster representation in Figure S5C). Insets show the zoomed-in view of ventricles or rosette-like structures. (F–H) Spatial transcriptomic landscape of CO (F), CO noMG with complex cytoarchitecture (G), and CO noMG with simple cytoarchitecture (H) colored by latent time projection from snRNA-seq samples. Insets show the zoomed-in view of one region of a ventricle (G) or rosette-like unit (H), showing the progression from progenitors to neuronal layers (arrow). Arrowheads in (F) indicate UL neurons. Note the scrambled temporal identity in CO noMG with small rosette-like units. (I) Heatmap displaying the expression of highly variable genes from the spatial transcriptomic landscape of CO (left) and CO noMG with simple cytoarchitecture (right), with cells ordered by latent time. Individual genes are highlighted as markers of specific cell clusters shown by color code. The top × axis color bar is cells colored by cell cluster identity. oRG, outer radial glia. Scale bars: (A and B) 200 μm and insets 20 μm.

The result was a map of spatial distribution of the selected transcripts with single-cell resolution (Figure 4A; Figure S5A). Several commonly used markers showed the expected distribution, validating the approach. For example, *SOX2* probe revealed VZ expression for day 55 CO organoid (Figure 4A; Figure S5A). We also analyzed CO noMG organoids with different morphologies, one with large ventricles and complex cytoarchitecture, and another with only small neural rosette-like structures and simpler cytoarchitecture (Figure 4B; Figure S5A). The predictive power of morphology was again evident as we found that despite the fact that the CO noMG organoids were treated identically to each other and even came from the same batch, the CO noMG organoid with simple cytoarchitecture displayed rather sparse RG markers (*PAX6*, *SOX2*, *FAM107A*, *MOXD1*, and *TNC*) spreading into neuronal regions, whereas in CO and CO noMG with proper ventricular structure, RG markers were appropriately restricted to the VZ (Figures 4A and 4B, insets; Figure S5A). This suggests an aberrant gene expression profile in space for organoids with simple cytoarchitecture. Further, *EOMES* (*TBR2*) transcript signal was nicely detected in the SVZ of CO but not in rosette-like ventricles of CO noMG (Figures 4A and 4B, insets; Figure S5A). Whereas both the DL neuron marker *BCL11B* and the UL neuron markers *SATB2*, *CUX1*, and *CUX2* were present in CO, only a few sparse *SATB2*+ UL neurons were observed in CO noMG with simple cytoarchitecture (Figure S5A, orange arrows) together with very limited spatial segregation between UL and DL neurons (Figures 4A and 4B, insets), validating the snRNA-seq comparisons showing depletion of UL neurons in LSM organoids (Figures 3H and 3I).

Next, we integrated the snRNA-seq landscape to leverage the information provided by the spatial data, together with the gene coverage provided by snRNA-seq. This enabled us to transfer annotations such as cell types from the snRNA-seq landscape (Figures 4C–4E). As expected, in CO and CO noMG with complex cytoarchitecture, RG cells mapped to the VZ, surrounded by IPCs in the SVZ (Figures 4C and 4D). Spatial segregation between DL and UL neurons was evident at this resolution in large ventricles (Figure 4D). By contrast, where no clear ventricles were distinguishable in CO noMG, RG cells were sparse (Figure 4E; Figure S5C), with very few IPCs (Figure 4E; Figure S5C), which were not located in an SVZ. INs were increased in CO noMG, consistent with our observations from snRNA-seq analysis (Figures 3B, 4D, and 4E; Figure S5C). These findings further support the notion that a complex tissue architecture and discrete ventricles are necessary for the full developmental repertoire of cell types and their spatial positioning.

### Tissue architecture influences fate progression

We next investigated temporal dynamics through pseudotime analysis. First, we applied RNA velocity as implemented in scVelo to estimate latent time progression, reflecting the course of cell differentiation. In concordance with *in vivo* development, neural progenitors were located at the root of the differentiation trajectories, followed by basal progenitors, migrating neurons, DL neurons, and lastly UL neurons, mature excitatory neurons, and INs (Figure S6A). Late-born INs seem to be among the last cell types to be generated, as seen *in vivo*.^37^

Next, we projected the latent time onto the spatial transcriptomic landscape (Figures 4F–4H). The resulting transcriptional dynamics in CO and CO noMG with complex cytoarchitecture were highly consistent with the known spatial directionality of the lineage *in vivo*, with the direction of tissue development starting from the VZ and terminating at the CP (Figure 4G, inset), with late maturing areas corresponding to late-born UL neurons (Figure 4F). However, these spatiotemporal trajectories were disrupted in CO noMG with simpler cytoarchitecture (Figure 4H, inset). Early progenitors were interspersed with late-born INs, and cells within rudimentary rosette-like structures showed mixed temporal identities. Thus, tissue architecture appears to be central for proper timed development of progenitors and neurons in organoids.

Following the observation of aberrant developmental trajectories in organoids with simpler tissue architecture, we looked at gene-wise expression dynamics across highly variable genes in the spatial transcriptomic landscape of CO and CO noMG (Figure 4I; Figure S6B). The developmental timing of expression for these genes can be represented as a function of projected latent time estimations across cells, and as such, it can be visualized using heatmaps. The heatmap for CO revealed a sharp transition from progenitor stage to neuronal stage, progressing through oRGs and IPCs (Figure 4I). Further neuronal maturation and IN development occurred later in this pseudotime. CO noMG with complex cytoarchitecture and discrete ventricles exhibited a developmental trajectory close to CO (Figure S6B). By contrast, CO noMG with simple cytoarchitecture showed differentiation dynamics lacking this timely progression of gene expression (Figure 4I). Progenitor markers such as *PAX6* continued to be expressed in neurons, and both UL and DL neuronal markers such as *SATB2* and *SOX5*, respectively, were expressed in progenitor cells during the same pseudotemporal period. Furthermore, the transition through oRGs and IPCs was lost, and GABAergic neuronal markers such as *GAD1*, *GAD2* appeared early, together with progenitor markers. These data strongly support the conclusion that complex tissue architecture and discrete ventricles enable a physiological progression of cell fate acquisition in cerebral organoids.

To further test this, we turned back to the snRNA-seq data, analyzed the differentially expressed genes from HSM and LSM organoids, and examined their developmental progression in latent time. Progenitor-associated differentially expressed genes were plotted along latent time. The overall gene expression trend associated with different morphologies revealed a similar pattern irrespective of the morphology (Figure S6C, left). However, LSM organoids lacked the basal progenitor transition phase observed for HSM, confirming our findings from the spatial landscape (Figure S6C, asterisk). Developmental progression for the neuronal counterpart (Figure S6C, right) showed an upregulation of progenitor genes still present in the neuronal population in LSM, whereas the HSM neuronal population behaved as expected with no expression of progenitor genes. This finding reinforced the idea that tissue morphology is linked to temporal fate.

### Perturbing spatial conformation disrupts temporal identity

**Figure 5:**
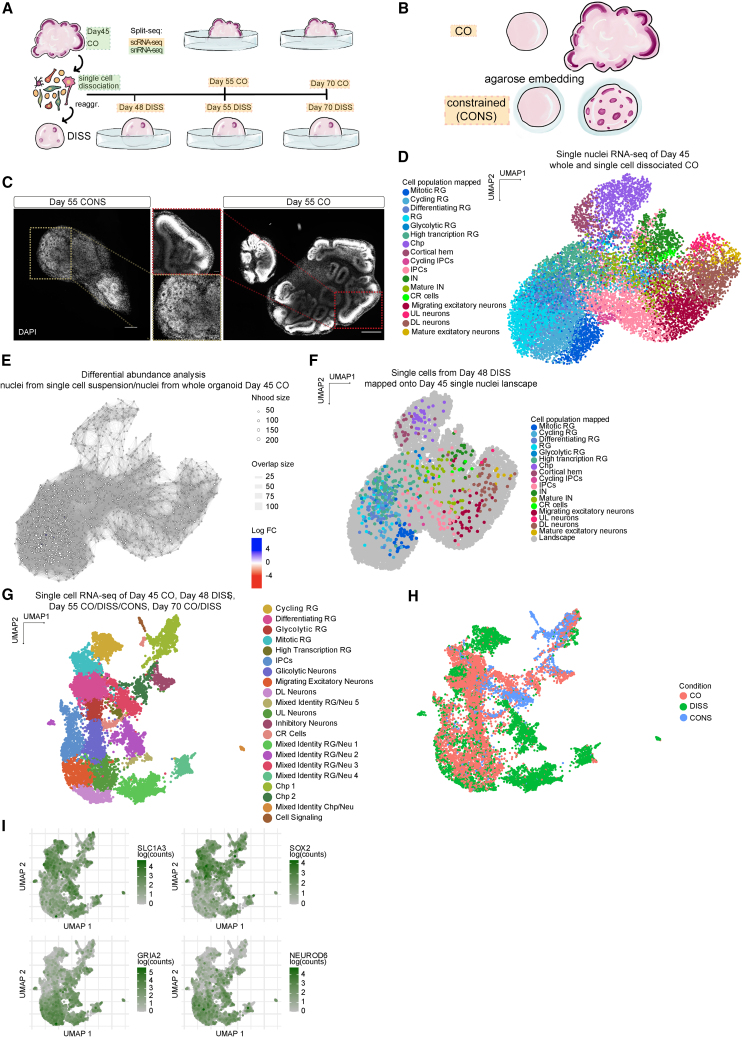
Perturbing spatial conformation disrupts temporal identity (A) Schematic of RNA-seq experiment. COs at day 45 were dissociated into single-cell suspension and reaggregated or their nuclei extracted for sequencing. In parallel, single nuclei were extracted from whole CO at day 45 and sequenced. Samples processed for snRNA-seq are highlighted in green. Single-cell suspension was plated to form several reaggregates followed over time. Dissociated-reaggregated organoids (DISS) were collected for single-cell RNA-seq at days 48, 55, and 70. In parallel, unperturbed COs were collected for sequencing at days 55 and 70. Two biological replicates per batch per time point across three batches were included, except for day 45 nuclei from single-cell suspension (2 biological replicates from 2 batches), single cells from day 48 (2 biological replicates per batch from 2 batches), and single cells from day 55 (2 biological replicates per batch from 2 batches). (B) Schematic of the strategy for constrained morphology organoid (CONS) by embedding in agarose hydrogel. (C) DAPI nuclear staining of sectioned day 55 CO and CONS organoids. Insets show small neural rosette-like structures in CONS versus large ventricles in CO. (D) UMAP plot of snRNA-seq landscape of day 45 whole organoids and day 45 single-cell suspension. Cells are color coded according to their identity. Cluster labels were transferred from the previously performed snRNA-seq shown in Figure 3. Two biological replicates across three batches for whole organoids and two samples from two different batches for single-cell dissociated organoids. (E) Differential abundance analysis of nuclei from CO day 45 single-cell suspension versus nuclei from whole CO day 45 performed with MiloR. The UMAP plot shows cell neighborhood (Nhood) connections using a shared nearest neighbor graph, highlighting over-represented (in blue) regions in the transcriptomic landscape. Nodes are neighborhoods, colored by their log-fold change across conditions. Non-differentially abundant (DA) neighborhoods are colored in white, and sizes correspond to the number of cells in a neighborhood. Graph edges depict the number of cells shared between adjacent neighborhoods. No differences were evident. FC, fold change; FDR, false discovery rate. (F) Projection of day 48 DISS cells onto day 45 single nuclei transcriptional landscape through nearest neighbor. Cell types were overall conserved in the organoid 3 days post reaggregation. (G) UMAP plot of scRNA-seq of CO day 45, DISS day 48, CO and DISS day 55, CO and DISS day 70, and CONS day 55 colored by cell type annotation. (H) UMAP plot color-coded by condition (CO, DISS, and CONS). (I) UMAP plot of RG markers (*SLC1A3* and *SOX2*) and neuronal markers (*GRIA2* and *NEUROD6*). Note the bivalent RG/neuron nature of mixed identity cell clusters. Scale bars: 500 μm, insets 100 μm in (C).

The correlation between aberrant morphology and dysmaturity in both snRNA-seq and spatial transcriptomics, regardless of the protocol applied, suggests the two may be causally linked. To more directly test whether tissue shape can drive temporal progression, we performed two different perturbations. First, we performed a set of new dissociation-reaggregation experiments to disrupt cytoarchitecture and followed their progression over time by single-cell RNA sequencing (scRNA-seq) (Figure 5A) to track the morphogenetic response longitudinally. Second, we applied a physical constraint in the form of hydrogel encapsulation to force organoids to take on a specific geometry and thus directly perturb morphology without affecting other aspects of the culture (Figure 5B). For the latter, healthy COs were encapsulated at day 13 in a low deformable, yet highly permeable and inert agarose hydrogel, thus enforcing a constrained morphology (CONS), followed by scRNA-seq at day 55 (Figure 5B). CONS organoids displayed a smooth surface with no protruding ventricles, instead displaying rosettes with small lumens and thin proliferative layers (Figure 5C). Thus, imposing a simplified morphology in this manner also led to simplified tissue architecture without influencing culture conditions, providing a tool to directly investigate the effect of morphology on cell fate. Encapsulated CONS organoids showed no significant signs of mitochondrial stress (*BCL2* and *BAX*), hypoxia (*HIF1A*), oxidative stress (*SOD1*), endoplasmic reticulum (ER) stress (*XBP1*), autophagy (*ATG5*), or apoptosis (*CASP8, 9*) when compared with CO (Figure S6D). To examine the effects of force exertion by the encapsulation itself, we looked at markers of compressive stress in CONS and CO. We assessed the expression of *VCL*, *VIM*, and *CDH1*, shown to be upregulated upon mechanical compression in 2D monolayer cells,^38^ and found no differences other than for *VIM*, which is also a marker of RG cells. On the other hand, we observed a mild upregulation of mechano-sensing genes: *TRPM3*, *TRPC1*, *TRPV4*,^39^ *NEAT1*,^40^ and *YAP1/TAZ*^41^ in CONS, validating the existence of a tissue response to mechanical encapsulation but no evidence of mechanical stress.

For the new set of dissociation-reaggregations (DISS), we collected organoids at day 45 upon initial dissociation, then 3 days post reaggregation (day 48), 10 days post reaggregation (day 55), and finally day 70 (Figure 5A). In addition, we included a set of control experiments to test whether differences in cell types post dissociation-reaggregation may in fact reflect differences in the survival of particular cell types. We sequenced nuclei extracted from whole organoids that were flash frozen at day 45, and nuclei from organoids dissociated at day 45 (Figure 5D). Nuclei from dissociated organoids showed no significant differences in cell type composition compared with nuclei from whole tissue in the transcriptional landscape, as shown by differential abundance analysis performed with MiloR (Figure 5E). Similarly, when DISS samples were analyzed 3 days post reaggregation, cell types matched the transcriptional profile of day 45 nuclei, suggesting that the mechanical perturbation itself did not impact the transcriptomic signature (Figure 5F). These data suggest that the dissociation and reaggregation of organoids represent a way of shuffling cellular position within the organoid, and thus perturbing architecture, without directly impacting cell fate.

Analysis of the scRNA-seq of DISS and CONS organoids compared with CO (Figure 5G) revealed that although CONS and DISS represent very different mechanical manipulations, they led to surprisingly similar effects on the transcriptional landscape. Both CONS and DISS samples displayed cells with an indistinct identity expressing progenitor and neuronal markers (mixed identity RG/Neu, Figures 5G and 5H). These cells clustered apart from the respective RG and neuronal clusters, indicating a signature different from bona fide RG and neurons but expressing both RG and neuronal genes, as shown by the concurrent expression of *SLC1A3*, *SOX2* (RG markers) and *GRIA2*, *NEUROD6* (neuronal markers) (Figure 5I).

**Figure 6:**
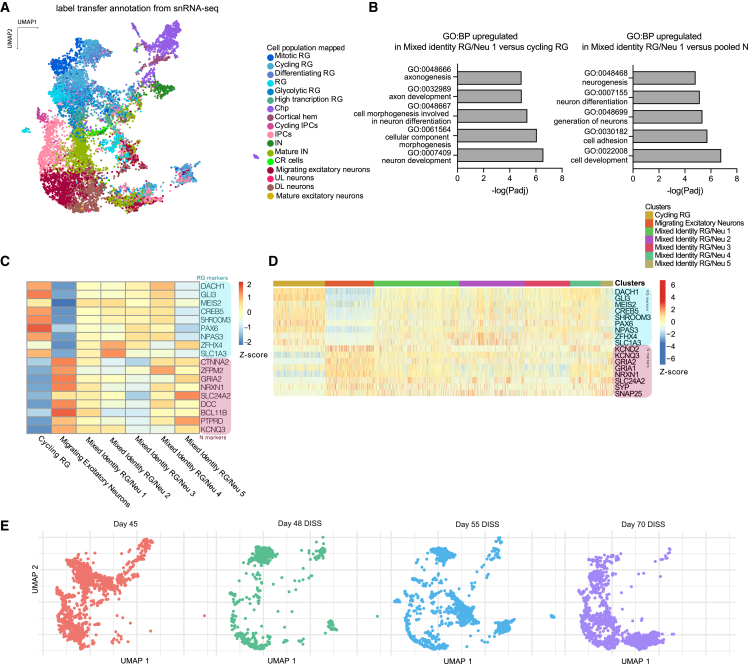
Cell mis-positioning results in mixed identity (A) UMAP plot colored by transferred cell type labels from previous snRNA-seq (Figure 3). Note clusters with mixed progenitor and neuronal identities. (B) Gene ontology (GO) term analysis showing upregulated GO:BP (biological process) terms for mixed identity RG/Neu cluster 1 compared with cycling RG cluster and the respective −log (adjusted p value) (left). GO term analysis showing upregulated GO:BP terms for mixed identity RG/Neu cluster 1 compared with pooled neuronal clusters (N) and the respective −log (adjusted p value) (right). (C) Z-scaled log(normalized counts) for RG and neuronal (N) marker genes plotted for mixed identity RG/Neu clusters, cycling RG, and migrating excitatory neuron clusters. RG and neuronal markers were manually selected according to the literature. (D) Single-cell values of Z-scaled log(normalized counts) for RG and neuronal markers plotted for mixed identity RG/Neu clusters, cycling RG, and migrating excitatory neuron clusters. RG and neuronal markers were manually selected according to the literature. (E) UMAP of DISS samples split by time point. Note the progression and continued presence of mixed identity clusters to day 70.

This mixed identity was further evident when cluster annotation was performed by cell type label transferring from our previously analyzed snRNA-seq dataset (Figure 6A), which revealed those same clusters containing a mixture of various identities. Furthermore, gene set enrichment analysis of differentially expressed genes between mixed identity cells and cycling RG as well as neurons, revealed significant differences in related biological processes. Mixed identity cluster 1 displayed a more mature signature when compared with cycling RG. Upregulated biological processes included terms associated with axon development and neuron differentiation. Contrarily, the same mixed identity cluster 1 appeared to be immature compared with neuronal clusters with upregulated biological processes related to neurogenesis (Figure 6B).

Mixed identity RG/Neu clusters showed a poised expression profile with both RG and neuronal markers (Figure 6C). These data suggest that these clusters were composed of cells with both immature and mature transcriptional signatures, indicating disrupted temporal progression. To inspect whether the mixed temporal identity of clusters present in morphologically perturbed organoids was due to individual cells progressing abnormally, we compared the expression of RG and neuronal markers from cells within progenitor and neuronal clusters, as well as the mixed identity clusters from DISS and CONS. Heatmap plots demonstrated that mixed identity RG/Neu 1, 2, and 4 (enriched in DISS) and mixed identity RG/Neu 3 (enriched in CONS) all displayed single cells expressing both RG and neuronal markers simultaneously (Figure 6D). The fact that such mixed identity cells were still present even in the more mature DISS day 70 (as shown by mixed identity RG/Neu 1) indicates that this state was maintained over an extended period of time (25 days) despite progenitor turnover, suggesting the tissue environment continued to impact cell fate progression (Figure 6E). Altogether, these data indicate that cells disrupted in space, following cytoarchitecture reshuffling or morphological perturbation, differentiated into cells with a disrupted identity in time.

## Discussion

The establishment of proper tissue architecture is heavily dictated by hard-wired genetic programs that direct cells to specific identities and positions, but how that same cellular positioning may impact cell fate programs is less clear. We took advantage of cerebral organoids to address this form-fate question in the context of the brain. Through comprehensive and semi-automated screening of organoid morphological features, we identified key protocol variations that impact tissue morphology and found that higher similarity to the *in vivo* transcriptome correlates with organoid morphology. In a recent transcriptome comparative study,^10^ it was reported that brain organoids diverge from *in vivo* in several ways, including a reduction in UL neurons and IPCs. We observed similar decreases in organoids with LSM. Thus, tissue shape could explain the differences in transcriptome and stress the importance of morphology in cerebral organoid studies.

We further investigated how the spatial organization may influence temporal progression. Latent time estimations were projected onto the spatial transcriptomic landscape, revealing disrupted temporal programs in organoids with simple cytoarchitecture and morphology. Further, directly perturbing tissue architecture through dissociation and reaggregation or tissue constraint resulted in conserved changes in transcriptome, with unique cell types with both progenitor and neuronal signatures. These direct morphological and cytoarchitectural perturbations thus resulted in a scrambling of not only cell position but also temporal identity. This supports the idea that tissue structure and cell fate are linked. The data together suggest that cells use their surroundings to interpret developmental time.

### Limitations of the study

There are intrinsic limitations of cerebral organoids as an *in vitro* model with issues such as batch-to-batch and intra-batch variability.^9^ Although our findings suggest higher reproducibility in guided organoids with patterning molecules, such reproducibility appears to be at the expense of a more complex tissue architecture. A fully reproducible method that maintains advanced morphology is still needed, but careful adjustment of cell seeding number, FGF2 concentration, and meticulous MG removal can result in unguided cortical organoids with an 80%–90% success rate.^42^ This study also demonstrates that tissue morphology reflects organoid similarity to *in vivo* and poses the foundation for a routine check of organoid quality. Nevertheless, the ultimate direction of this correlation is yet to be determined. *A priori* variations in gene expression across samples could also account for morphogenetic differences. Although this study demonstrates a link between shape and fate in cerebral organoids, the bidirectional cross-talk between the two makes it challenging to disentangle one from the other. Nevertheless, a similar response to different morphological and structural perturbations points to a conserved mechanism regardless of the manipulation performed. Although the shared signature is not likely to arise from cell stress, we could not exclude a contribution of force exertion or tissue stiffness. Finally, the study did not examine the mechanistic aspect of the relationship between tissue shape and fate, and further studies are needed to investigate this.

## STAR★Methods

### Key resources table

**Table:** 

REAGENT or RESOURCE	SOURCE	IDENTIFIER
**Antibodies**		

Rabbit anti-SOX2	Abcam	Cat#ab97959, RRID:AB_2341193
Mouse anti-HuC/D	Invitrogen	Cat#A21271, RRID:AB_221448
Sheep anti-TBR2	R&D Systems	Cat#AF6166, RRID:AB_10569705
Rat anti-BCL11B	Abcam	Cat#ab18465, RRID:AB_2064130
AlexaFluor 568, 647 donkey anti-sheep IgG (H+L)	Life Technologies	Cat#A21099, RRID: AB_2535753; Cat#A21448, RRID: AB_2535865
AlexaFluor 488, 568, 647 donkey anti-mouse IgG (H+L)	Life Technologies	Cat#A21202, RRID: AB_141607; Cat#A10037, RRID: AB_2534013; Cat#A31571, RRID: AB_162542
AlexaFluor 488, 568, 647 donkey anti-rabbit IgG (H+L)	Life Technologies	Cat#A32790, RRID: AB_2762833; Cat#A10042 RRID: AB_2534017; Cat#A31573, RRID: AB_2536183
AlexaFluor 488 donkey anti-rat IgG (H+L)	Life Technologies	Cat#A21208, RRID:AB_2535794

**Chemicals, peptides, and recombinant proteins**		

StemFlex	Thermo Fisher	Cat#A3349401
Matrigel	Corning	Cat#356234
Accutase	Sigma-Aldrich	Cat#A6964
ROCK inhibitor Y27632	Millipore	Cat#SCM075
FGF2	Peprotech	Cat#100-18B
SB 431542	Sigma-Aldrich	Cat#S4317
Dorsomorphin	Sigma-Aldrich	Cat#P5499
IWR1endo	Stratech	Cat#S7086-SEL
CHIR 99021	Tocris	Cat#4423
EGF	R&D systems	Cat#236-EG
Low gelling temperature agarose	Sigma	Cat#A9414
Accumax	Sigma	Cat#A7089
DNAse	Sigma	Cat#04716728001
PAXgene fixative	QIAGEN	Cat#765312
Tissue STABILIZER	QIAGEN	Cat#765512
ProLong Diamond	ThermoFisher	Cat#P36961
Tris-HCl	Invitrogen	Cat#AM9856
MgCl2	Invitrogen	Cat#AM9530G
NaCl	Sigma	Cat#71386
Nonidet P40 substitute	Roche	Cat#11754599001
protector RNase inhibitor	Sigma	Cat#3335399001
Halt protease inhibitor	ThermoFisher	Cat#1860932
DTT	Sigma	Cat#646563
Bovine Serum Albumin	ThermoFisher	Cat#15260037
Accumax	Merck	Cat#A7089
Fetal bovine serum	Merck	Cat#F2442
DMEM F-12	Thermo Fisher Scientific	Cat#11330032
Neurobasal Medium	Invitrogen	Cat#21103049
MEM-NEEA	Sigma	Cat#M7145
GlutaMAX	Thermo Fisher Scientific	Cat#35050038
Penicillin-Streptomycin	Sigma	Cat#P0781
N2 supplement	Thermo Fisher Scientific	Cat#17502048
B27 supplement	Thermo Fisher Scientific	Cat#17504044
B27 minus vitamin A	Thermo Fisher Scientific	Cat#12587010
β-Mercaptoethanol	Life Technologies	Cat#31350-010
Knockout Serum Replacement	Thermo Fisher Scientific	Cat#10828028
Insulin	Sigma	Cat#I9278
Heparin	Sigma	Cat#H3149
DAPI	Life Technologies	Cat#D1306
OCT	VWR Chemicals	Cat#361603E
2-methylbutane	Sigma	Cat#M32631

**Critical commercial assays**		

STEMdiff™ Cerebral Organoid Kit	StemCellTechnologies	Cat#08570
Evercode (WT and WT Mini) v3	Parse Biosciences	N/A
Single Cell 3′ Library & Gel Bead Kit v3	10x Genomics	N/A
Oligonucleotide Probes for Molecular Cartography	Resolve Biosciences	See Methods S1

**Deposited data**		

Raw snRNA-seq and scRNA-seq data	This paper	GEO: GSE241543 [NCBI tracking system #24229782]
Processed snRNA-seq data	This paper	https://cells-test.gi.ucsc.edu/?ds=brain-org-morph
RNA-seq code	This paper	https://github.com/rosshandler/Structure-Indentity.https://doi.org/10.5281/zenodo.8322350
Fiji/ImageJ code for morphological screening	This paper	https://github.com/jboulanger/Organoid_morphologyhttps://doi.org/10.5281/zenodo.8322334

**Experimental models: Cell lines**		

Human ESCs H9	WiCell	WA09

**Software and algorithms**		

Morphological analysis of organoid brightfield images	Fiji/ImageJ	https://github.com/jboulanger/imagej-macro/tree/main/Contour_Curvature
Fiji/ImageJ	Schindelin et al.^43^	https://imagej.net/Fiji
Prism v9.5.1	GraphPad	https://www.graphpad.com/
Python v3.9	Van Rossum, Guido and Drake, Fred L. 2009^44^	https://www.python.org/
R Studio v2023.06.0+421	R Core Team, 2018^45^	http://www.R-project.org/
g:Profiler	Raudvere et al.^46^	https://biit.cs.ut.ee/gprofiler/gost
Jellyfish	Marçais and Kingsford^47^	N/A
ThermonucleotideBLAST	Gans and Wolinsky^48^	N/A
APPRIS	Rodriguez et al.^49^	N/A
Iterative closest point algorithm	Resolve Biosciences	https://github.com/ethz-asl/libpointmatcher
Polylux plugin	Resolve Biosciences	N/A
QuPath v0.3.0	Bankhead et al.^50^	N/A
CellRanger_3.1.0	10x Genomics	N/A
Velocyto_0.17.17	La Manno et al.^51^	N/A
Scran_1.20.1 R	Lun et al.^48^^,^^49^	N/A
Seurat_4.0.3	Satija et al.^52^	N/A
ScVelo_0.2.4	Bergen et al.^53^	N/A
Spipe v.1.0.3	Parse Biosciences	N/A

### Resource availability

#### Lead contact

Further information and requests for resources and reagents should be directed to and will be fulfilled by the lead contact, Madeline Lancaster (madeline.lancaster@mrc-lmb.cam.ac.uk).

#### Materials availability

• Probes generated for Molecular Cartography have been deposited to Resolve Biosciences with the respective catalogue number and ENSEMBL IDs as listed in Methods S1 table.

### Experimental model and study participant details

#### Cell lines

Human ESCs (H9, female) were used in this study. H9 (WA09) was purchased from WiCell. Human ESCs used in this project were approved for use in this project by the UK Stem Cell Bank Steering Committee and approved by an ERC ethics committee and are registered on the Human Pluripotent Stem Cell Registry (hpscreg.eu). Cells were maintained in StemFlex (Thermo Fisher, A3349401) on Matrigel (Corning, 356234) coated plates at 37 °C with controlled 5% CO2. Cells were passaged every 3-4 days using 0.7 mM EDTA. Cells used for experiments were exclusively under passage 50. Commercial cell line H9 was authenticated by the provider.

### Method details

#### Organoid generation

Basal cerebral organoids (bCOs) were generated as previously described^15^; briefly single cell suspension was obtained through resuspension in Accutase (Sigma-Aldrich, A6964). 2000 cells were seeded in U-bottom ultralow attachment 96 well plates (Corning, CLS7007) to generate embryoid bodies (EBs) in hESC medium (400 ml of DMEM-F12 (Thermo Fisher Scientific, 11330032), 100 ml of KOSR (Thermo Fisher Scientific, 10828028), 15 ml of ESC-quality FBS (Merck, F2442), 5 ml of GlutaMAX (Thermo Fisher Scientific, 35050038), 5 ml of MEM-NEAA (Sigma, M7145) and 3.5 μl of β-mercaptoethanol (Life Technologies, 31350-010) were combined)^26^ and supplemented with 50μM ROCK inhibitor Y27632 (Millipore, SCM075) and 4ng/mL bFGF (FGF2, Peprotech 100-18B) from day 0 to day 3. dS bCOs were generated as bCOs but were additionally supplemented from day 1 with 10μM SB 431542 (Sigma-Aldrich, S4317) and 1μM Dorsomorphin (Sigma-Aldrich, P5499) until day 5. SW bCOs were generated as bCOs but were additionally supplemented from day 0 with 10μM SB 431542 (Sigma-Aldrich, S4317) and 3μM IWR1endo (Stratech, S7086-SEL) until day 5. From day 5 bCO, dS bCO, and SW bCO were treated identically and the medium was replaced with neural induction medium (DMEM-F12 with 1% (vol/vol) N2 supplement (Thermo Fisher Scientific, 17502048), 1% (vol/vol) GlutaMAX supplement and 1% (vol/vol) MEM-NEAA, heparin (Sigma-Aldrich, H3149) at a final concentration of 1 μg/ml)^26^ and refreshed every two days until day 11. At day 11 EBs were embedded in Matrigel droplets (Corning, 356234) as described previously^26^ and maintained in neural induction medium until day 13. At day 13 organoids were transferred to improved differentiation medium without vitamin A (for 250 ml of medium, 125 ml of DMEM-F12, 125 ml of Neurobasal medium (Invitrogen, 21103049), 1.25 ml of N2 supplement (Thermo Fisher Scientific 17502048), 62.5 μl of insulin (Sigma-Aldrich, I9278), 2.5 ml of GlutaMAX supplement, 1.25 ml of MEM-NEAA and 2.5 ml of penicillin-streptomycin, 87.5 μl of 1:100 dilution of β-mercaptoethanol in DMEM-F12 were combined. 2.5 ml of B27 minus vitamin A supplement (thermo Fisher Scientific, 12587010) were added) (IDM-A).^26^ bCOs, dS bCOs, SW bCOs were treated with the following conditions at day 13 and 15: untreated, +EGF/+FGF/+CHIR, +EGF/+FGF, +CHIR in IDM-A. CHIR 99021 (Tocris, 4423) was used at 3μM, EGF (R&D systems 236-EG) 10 ng/ml, bFGF (FGF2, Peprotech 100-18B) 10ng/ml. Matrigel was then removed manually with a fine scalpel and at day 18 organoids were transferred to an orbital shaker (φ25 cm) at 57 rpm in 5 cm tissue culture dishes. Organoids were fed with IDM+A with 1 ml dissolved Matrigel per 50 ml media^22^ from day 20. A commercial kit (STEMdiff™ Cerebral Organoid Kit, StemCell Technologies 08570) was used to generate optimized Cerebral Organoids (COs) using 2000 cells to generate EBs and supplementing with 50μM ROCK inhibitor from day 0 to day 3. From day 18 COs followed the same protocol as for bCOs. Unembedded organoids (COs noMG) were generated as above with the STEMdiff™ Cerebral Organoid Kit. However, organoids were neither embedded in Matrigel nor exposed to dissolved Matrigel in IDM+A. From day 7 to 18 unembedded organoids were kept in low attachment 24 well plates. Fast agitation (FAST) was achieved by increasing the orbital shaker (φ25 cm) speed to 78 rpm on COs in 5cm dishes from day 18. For further details see Tables S1 and S2.

#### Organoid encapsulation in agarose

To generate constrained (CONS) organoids, Matrigel was removed from Day 13 COs as above and embedded in 3% low gelling temperature agarose (Sigma-Aldrich, A9414) solution in sterile DPBS. Agarose constrained organoids were further processed in the same way as COs. 3 independent batches of organoids were collected at day 55 for sequencing.

#### Organoid dissociation-reaggregation

Day 45 COs generated with the STEMdiff™ Cerebral Organoid Kit and with a healthy morphology were dissociated using 1 ml of Accumax (Sigma-Aldrich, A7089) with 400 μg/ml DNAse (Sigma-Aldrich, 04716728001) per organoid by incubating at 37°C for 20 minutes with agitation and intermittent pipetting every 5 minutes. Cell suspension was then filtered using a 70 μm cell strainer (Corning, 352350). 25,000 to 100,000 cells were then plated in a well of U-bottom ultralow attachment 96 well plate (Corning, CLS7007), in IDM+A+dissolved Matrigel. The plate was then briefly and gently centrifuged at 40xg for 2 minutes before placing in the incubator. 3 days later, aggregates were collected using a cut P1000 pipette tip and processed for sequencing or transferred to 5 cm dishes with IDM+A+MG and placed back on the shaker until collection at day 55 and day 70. Biological replicates (batches) were reaggregates followed over time at day 48, day 55, and day 70 that were derived from the same single cell suspension obtained from organoids within the same batch at day 45. Thus, sample-to-sample variability was significantly reduced when analysing organoid development over time.

#### Morphometric analysis

Organoids were imaged throughout their development using an Evos XL Core (Thermo Fisher Scientific) in brightfield mode. TIFF pictures were processed in Fiji/ImageJ with the custom-designed macro available at (https://github.com/jboulanger/imagej-macro/tree/main/Contour_Curvature). The parameters measured are listed below. The region of interest (ROI) encompassing the outline of the organoid was traced manually. At day 3, six batches of 1-10 organoids per batch per condition were analysed, with the exception of CO (five batches). At day 5, six batches of 1-10 organoids per batch per condition were analysed, with the exception of dS bCO and CO (five batches each). At day 18, six batches of 1-10 organoids per batch per condition were analysed, with the exception of dS bCO and CO (five batches). For PCA plot showing averaged data from day 3 to day 18 (Figure 1D) each morphological value was averaged across organoids within each batch. For day 55 organoids, the same macro was used on the same organoids processed for snRNA-seq at day 55 (Batch A: CO, FAST, DISS, CO noMG; Batch B: CO, FAST, DISS; Batch C: CO, FAST, DISS, CO noMG_a, CO noMG_b; images shown in Figure S4F), but transparency was not measured due to the large size of the organoid. Principal component analysis was performed using the function prcomp in R. The importance of morphological variables across the three top principal components (dimensions) was assessed by the squared cosine and plotted as a correlation plot.

**Table:** 

Morphological features
- Area: area of selection in μm^2^ - Perimeter: length of the outside boundary of the selection in μm - Feret’sdiameter: the longest distance between any two points along the selection boundary - Roundness: 4 x [Area/π (Major axis)^2^] - Circularity: 4π x [(Area/Perimeter^2^)] - Inflection points: number of inflection points in the contour of the shape - Dirichlet Normal Energy (DNE) x R0: logarithm of the square of the variation of the normal n=(dy,-dx) of the contour projected on its tangent t=(dx,dy) where dx and dy are the first derivative in x and y.^54^^,^^55^ DNE is normalised by the average radius (R0). - Standard Deviation of the Curvature (StdCurvature) x R0: standard deviation of the logarithm of the sum of the square of the curvature of the contour. Where the curvature along the contour is the inverse of the radius of the tangent circle and is computed as [(dx ^∗^ dyy) – (dy ^∗^ dxx)] / [(dx^∗^dx) + (dy^∗^dy)] ^3/2^ where dx and dy are the first derivative in x and y and dxx and dyy are the second derivative of the contour. The standard deviation of the curvature is normalised by the average radius (R0). - Transparency: the mean response of the Laplacian of Gaussian (LoG) filter. The more the object is transparent the more textured it becomes and thus the higher the filter values.

For statistical comparison between brightfield morphological measurements and macro architecture of organoid sections, surface complexity parameters as measured from brightfield pictures (Circularity, Inflection points, StdCurvature x R0) were correlated to macro architecture features for the same organoids (ventricle number, ventricle length) by calculating the Spearmann correlation coefficient. *P* values<0.05 from multiple comparisons were indicated in bold in the figure (Figure 1M). Two-tailed nonparametric Spearman correlation was performed. 7 organoids from one batch were analysed. Single ventricles were measured and plotted as average value per organoid. Number of ventricles measured varied from 1-18 per organoid.

#### Organoid processing for immunohistochemistry

At day of fixation (day 18, day 55) organoids were washed in Phosphate Buffer Saline (PBS) and incubated in 4% PFA in phosphate buffer (PB) for 1 hour at room temperature (RT). After fixation organoids were washed in PBS and submerged in 30% sucrose/0.02% Sodium Azide (Sigma, S2002) in PB at 4°C until the tissue sank. The tissue was then embedded in 7.5% gelatin/30% sucrose in PB and frozen in a cold isopentane bath (Sigma, 277258) at -50°C; tissue blocks were stored at -80°C until sectioning. Blocks were sectioned using a cryostat (Leica, CM1950) at 20 μm and sections were collected on charged slides (ThermoFisher Superfrost Plus, J1800AMNZ) and stored at -20°C until further processing.

#### Immunohistochemistry (IHC)

Slides with cryosections were rinsed in PBS for 15 minutes. Slides were incubated in permeabilization buffer (0.25% Triton-X 100 with 4% donkey serum in PBS) for 1h at RT. Primary antibodies were diluted in blocking buffer (0.1% Triton-X 100 with 4% donkey serum in PBS) at different concentrations: anti-SOX2 1:200 (Abcam, ab97959), anti-HuC/D 1:500 (Invitrogen, A21271), anti-TBR2 1:300 (R&D Systems, AF6166), anti-BCL11B 1:500 (Abcam, ab18465). Primary antibody incubation was performed overnight at 4°C. Following washes in PBS, slides were incubated with secondary antibodies Alexa Fluor fluorescent dyes 1:1000 (Life Technologies) and DAPI 1:1000 in blocking buffer. Sections were incubated for 1-2h at RT. After PBS washes, slides were mounted in ProLong™ Diamond (Thermo Fisher Scientific, P36961) and stored at 4°C.

#### Imaging

Images were acquired either on a Pannoramic structured illumination based confocal slide scanner from 3DHISTECH with a 20x/0.8 NA Plan-Apochroma Zeiss air objective and a PCO edge CMOS camera or on LSM780 confocal inverted microscope from Zeiss with a 20x/0.8 NA Plan-Apochroma Zeiss air objective. Images were single planes. Images were analysed in Fiji/ImageJ.^56^

#### Quantification of IHC images

Ventricle length was measured by manually tracing the lumen perimeter, meaning the continuous line connecting the apical surface of the ventricle of peripherical ventricles of day 55 organoids using Fiji/ImageJ. CO=5 batches, CO noMG=3 batches, FAST=3 batches. Organoids per batch: 1-5; n=single ventricles. Alongside ventricle length, ventricle number was measured.

VZ and SVZ thickness were measured for day 55 organoids, plotted per ventricle and according to the ventricle length bin. VZ and SVZ were measured only for ventricles in the periphery of the organoid. VZ and SVZ thickness were determined according to TBR2 signal. VZ extended from the apical surface to the beginning of the TBR2+ cell layer. SVZ was measured as the thickness of the TBR2+ cell layer. VZ and SVZ thickness was always measured at the centre of the ventricle. Measurements were performed on Fiji/ImageJ. CO=5 batches, CO noMG=3 batches, FAST=3 batches. Organoids per batch: 1-5; n=single ventricles. Two-way ANOVA and Tukey’s multiple comparison test with a single pooled variance were performed using GraphPad Prism.

TBR2 or HuC/D staining intensity and distribution was captured by measuring mean gray intensity of the organoid ROI on Fiji/ImageJ and then normalised on the ROI area. N=single organoids from 3 batches per condition. Kruskal-Wallis test and Dunn’s multiple comparisons test were performed using GraphPad Prism.

#### Organoid processing for Molecular Cartography

Molecular cartography was performed according to Resolve Biosciences guidance. Organoids were fixed in PAXgene fixative (QIAGEN ID: 765312) for 2 hours at RT. Fixative solution was replaced with tissue STABILIZER (QIAGEN ID: 765512) for 3 hours and 30 minutes at RT. Tissues were dehydrated in 30% sucrose solution [w/v] over night at 4°C. Tissues were embedded in pre-cooled OCT (VWR Chemicals, 361603E) on dry ice and snap froze in isopentane at -50°C. Frozen samples were stored at -80°C before further processing. Organoids were sectioned with a cryostat (Leica, CM1950) to 10μm thickness. Sections were placed within the placement areas of cold Resolve Biosciences slides and stored at -80°C.

#### Molecular Cartography

Tissue sections were thawed and treated with isopropanol for 1 minute followed by one-minute wash in 95% ethanol and 70% ethanol at room temperature. Samples were used for Molecular Cartography (100-plex combinatorial single-molecule fluorescence in-situ hybridization) according to the manufacturer’s instructions (protocol 4.0; available for download from Resolve’s website to registered users), starting with the aspiration of ethanol and the addition of buffer BST1 (step 6 and 7 of the tissue priming protocol). Briefly, tissues were primed followed by overnight hybridization of all probes specific for the target transcripts (see below for probe design details and target list). Samples were washed the next day to remove excess probes and fluorescently tagged in a two-step process.^57^ Regions of interest were imaged as described below and fluorescent signals removed. Tagging and removal of fluorophores were repeated for a total of eight cycles to build a unique combinatorial code for every target transcript.

#### Probes Design

The probes for 100 transcripts were chosen from the literature to represent cell-specific selective markers known to be expressed in brain organoids or developmental human brain. The probes for 100 genes were designed using Resolve’s proprietary design algorithm. Briefly, for every targeted gene all full-length protein coding transcript sequences from the ENSEMBL database were used as design targets if the isoform had the GENCODE annotation tag ‘basic’.^58^^,^^59^ To reduce the calculation of computationally expensive steps like the off-target searches, the selection of probe sequences was limited to sequences with high success rates. Highly repetitive regions were filtered by obtaining the abundance of k-mers from the background transcriptome using Jellyfish.^43^ All target sequences were scanned for all k-mers and regions with rare k-mers preferred as seeds for full probe design. A probe candidate was generated by extending a seed sequence until sufficient target stability was reached. A set of simple rules was applied to discard sequences that were found experimentally to cause problems. After these fast screens, every kept probe candidate was mapped to the background transcriptome using ThermonucleotideBLAST^60^ and probes with stable off-target hits were discarded. Specific probes were then scored based on the number of on-target matches to isoforms, which were weighted by their APPRIS level,^61^ favouring principal isoforms over others. A bonus was added if the binding-site was inside the protein-coding region. The highest scoring probes for each transcript were used in the experiment. A list of gene names, ENSEMBL IDs and Resolve Catalogue numbers for the probes used for Molecular Cartography is listed in Methods S1.

#### Imaging for Molecular Cartography

Samples were imaged on a Zeiss Celldiscoverer 7, using the 50x Plan Apochromat water immersion objective with an NA of 1.2 and the 0.5x magnification changer, resulting in a 25x final magnification. Standard CD7 LED excitation light source, filters, and dichroic mirrors were combined with customized emission filters and images recorded with a Zeiss Axiocam Mono 712. Excitation time per image was 1000 ms for each readout channel (20 ms for DAPI). A z-stack was taken at each region with a 300 nm distance between z-planes. For each region, a z-stack per fluorescent colour (two colours) was imaged per imaging round. The 8 imaging rounds resulted in 16 z-stacks per region. Imaging of each round was fully automated via a custom python script using the scripting API of the Zeiss ZEN software (Open application development). Automated imaging included water immersion, focusing and precise sample positioning to capture the same image over multiple rounds. The protocol selectively detected human transcripts and not mouse-specific transcript Nlrp1a, which was used as a negative control.

#### Molecular Cartography Image Analysis

##### Spot Segmentation

The algorithms for signal segmentation were written in Java and are based on the ImageJ library functionalities.^51^ Only the iterative closest point algorithm is written in C++ based on the libpointmatcher library (https://github.com/ethz-asl/libpointmatcher).

##### Preprocessing

All images were corrected for background fluorescence. A target value for the allowed number of maxima was determined based upon the area of the slice in μm² multiplied by the factor 0.5. The brightest maxima per plane were determined, based upon an empirically optimized threshold. Maxima were independently identified for every image slice and maxima that did not have a neighbouring maximum in an adjacent slice (called z-group) were excluded. The resulting list was further filtered in an iterative loop by adjusting the allowed thresholds for (Babs-Bback) and (Bperi-Bback) to reach a feature target value (Babs: absolute brightness, Bback: local background, Bperi: background of periphery within 1 pixel). This feature target values were based on the volume of the 3D-image. Only maxima still in a z-group of at least 2 after filtering were passing the filter step. Each z-group was counted as one hit. The members of the z-groups with the highest absolute brightness were used as features and saved as a 3D-point cloud.

##### Decoding

To align the raw data images from different imaging rounds, images had to be corrected. To do so the extracted feature point clouds were used to find the transformation matrices. An iterative closest point cloud algorithm was used to minimize the error between two point-clouds. The point clouds of each round were aligned to the point cloud of round one - the reference point cloud. Based upon the transformation matrices the corresponding images were processed by rigid transformation using trilinear interpolation. The aligned images were used to create a profile for each pixel consisting of 16 values derived from the two imaged channels across 8 rounds. The pixel profiles were filtered for variance from zero normalized by total brightness of all pixels in the profile. Matched pixel profiles with the highest score were assigned as an ID to the pixel. Pixels with neighbours having the same ID were grouped and filtered by group size, number of direct adjacent pixels in group, number of dimensions with size of two pixels. The local 3D-maxima of the groups were determined as potential final transcript locations. Maxima were filtered by number of maxima in the raw data images where a maximum was expected. Remaining maxima were further evaluated by the fit to the corresponding code. The coordinated of the remaining maxima were written to the results file. This file now contains the x-, y- and z-coordinates of decoded transcripts and was used for further analysis. The ratio of signals matching to codes used in the experiment and signals matching to codes not used in the experiment were used to estimate specificity (false positives).

##### Downstream Analysis

Final image analysis was performed in ImageJ2^51^ using the Polylux plugin provided by Resolve BioSciences to examine specific Molecular Cartography signals.

##### Generation of gene-by-cell matrices

Cell segmentation was based on maximum projections of the DAPI images generated in the final imaging round of Molecular Cartography. Images were imported into QuPath v0.3.0,^62^ DAPI signals detected and expanded by 8 μm. Cell boundaries were exported as ImageJ ROI files and imported into ImageJ2. All transcripts overlapping with the single cell ROIs were assigned to the corresponding cell and exported as tab-delimited gene-by-cell matrices with Polylux.

#### Single nuclei dissociation

Day 45, 55 whole organoids or organoid slices 300 μm in thickness were snap froze in liquid nitrogen and stored at -80°C until further usage. Single nuclei isolation protocol was adapted from Frankestein protocol^63^^,^^64^^,^^65^^,^^66^ and Denisenko et al.^67^ Samples were transferred to a 1.5 mL tube containing 500 μL of chilled lysis buffer (10 mM Tris-HCl (Invitrogen, AM9856), 3 mM MgCl2 (Invitrogen, AM9530G), 10 mM NaCl (Sigma-Aldrich, 71386), 0.005% Nonidet P40 substitute (Roche, 11754599001), 1:400 protector RNase inhibitor 40U/μL (Sigma-Aldrich, 3335399001), 1:100 Halt protease inhibitor (ThermoFisher, 1860932), 1:10000 1M DTT (Sigma-Aldrich, 646563) in nuclease free water) and incubated on ice for 2 minutes. The tissue was then completely homogenized with a pellet pestle using up and down strokes. The full volume was then transferred to a pre-cooled 15 mL tube with 2 mL of lysis buffer. The homogenate was incubated on ice for 12 minutes and mixed with a glass pipette. Following the incubation, 2.5 mL wash buffer was added to the homogenate, which was subsequently filtered through a 30 μm strainer into a pre-cooled FACS tube. The samples were centrifuged at 500g for 5 minutes at 4°C. The supernatant was discarded. 1 mL of wash buffer (1xDPBS, 1% BSA (Thermo Fisher Scientific, 15260037), 1:400 protector RNase inhibitor 40U/μL) was then added to the pellet and resuspended. The wash was repeated a second time. The samples were centrifuged for the third time, the supernatant was aspirated and 500μl of wash buffer with 1:1000 DAPI were added. The samples were incubated on ice for 10 minutes. A quality control step was performed by viewing the nuclei under a fluorescence microscope to check nuclei shape and count. A Synergy Sorter was then used to sort DAPI-positive events using a 70μm nozzle and a pressure of 22 psi. Nuclei were collected in Nuclei Buffer (10x Genomics, PN-2000153, PN-2000207 with 1mM DTT, 1U/μL protector RNase inhibitor) and processed immediately on the 10x Chromium controller or fixed according to the Parse Biosciences protocol for nuclei fixation for further split-seq.

#### Single cell dissociation and Split-seq

Single organoids were incubated in 1 mL Accumax solution (Merck, A7089) supplemented with DNase I (Sigma, 04716728001) 1.25 U/mL. Organoids were incubated in the solution at 37°C for 20 minutes on a shaker followed by gentle agitation and pipetting. The enzymatic activity of Accumax was stopped by adding IDM+A or FBS (fetal bovine serum Merck, F2442) 1:10. The solution was filtered through 70μm cell strainer prior to cell counting. The solution was centrifuged at 200g for 10 minutes at 4°C and the pellet resuspended in cell prefixation buffer (Parse Biosciences). Sample fixation and split-seq were performed according to the Evercode Fixation v3 Parse Biosciences protocol. COs were collected at day 45, 55, 70 for sequencing. DISS organoids were collected for sequencing at day 48, 55, 70, and CONS organoids were collected at day 55. Parse Biosciences Evercode whole transcriptome WT was used for CO, CONS, and DISS single cells.

For nuclear split-seq, nuclei were isolated as described above, and sample fixation and split-seq were performed according to the Evercode Fixation v3 Parse Biosciences protocol for single nuclei. Nuclei were sequenced with Parse Biosciences Evercode whole transcriptome WT Mini. 20,000 total nuclei and 60,000 total cells were loaded for split-seq. Single cells were sequenced on a S2 Novaseq lane (4 billion reads depth). Single nuclei were sequenced on a SP Novaseq lane (400 million reads depth). N=2 organoids per batch (3 batches) per condition per time point exception made for Day 48 (2 batches), Day 55 CO (5 batches), Day 55 DISS (2 batches), Day 55 CONS (3 batches, 5 organoids).

#### Single nuclear droplet-based RNA-seq

20,000 nuclei per sample were processed for single nuclei droplet RNA-seq (10x Genomics). Single cell RNA-seq libraries were prepared according to manufacturer’s instructions using the 10x Genomics Chromium Single Cell 3’ Library & Gel Bead Kit v3 (10x Genomics) workflow. The Chromium Controller produced the single cell-bead droplets. Reverse transcriptase reaction and subsequent amplification was carried out on a C1000 Touch Thermal Cycler (Biorad). Libraries were quality tested using a 2100 Bioanalyzer Instrument (Agilent). Samples were sequenced in two rounds on one lane each of SP flowcell of the Novaseq6000 sequencer (Illumina). N=1 organoid per batch (3 batches) per condition except for CO noMG (2 batches 3 organoids).

#### Quality control analysis and normalization of droplet snRNA-seq data

Raw sequence reads were aligned using CellRanger_3.1.0 against the Human genome GRCh38-1.2.0 as provided by 10x Genomics. Intronic and exonic raw counts were extracted using velocyto_0.17.17,^47^ reads mapping to highly repetitive regions in the genome were excluded. The overall count matrix was obtained by adding both, pre-mRNA and mRNA UMIs. Cell libraries with low complexity (fewer than 500 expressed genes) were excluded. Next, cells with high mitochondrial UMI content were removed, as this is a known indicator of cell stress. In order to set a suitable threshold of mitochondrial read fraction for this dataset, a normal distribution centered on the median, with variance estimated using the median absolute deviation (MAD) was used to build a null model for computing a p-value for each cell library. A Benjamini–Hochberg (BH) adjusted p-vaue<0.05 was considered for rejection. The following steps were carried out using the scran_1.20.1 R package.^48^^,^^49^ Transcriptome size factors were calculated with ‘computeSumFactors’. Cells were pre-clustered with the ‘quickCluster’ function using the parameter ‘method = igraph’, and minimum and maximum cluster sizes of 100 and 3,000 cells, respectively. Raw counts for each cell were divided by their size factors, and the resulting normalized counts were used for further processing. The method ‘scDblFinder’ was applied to score cells for their doublet state in a sample-wise manner, because only cells from the same sample can form a doublet (where a sample is a single lane of a 10x Chromium chip). Then, a batch corrected PCA manifold was generated with the R package Seurat_4.0.3.^52^^,^^68^^,^^69^^,^^70^ For each sample, library size normalization and variance stabilizing transformation (vst) were applied. The top 2,000 highly variable genes were then extracted for the integration step and reciprocal PCA (‘method = rpca’) was used to find the corresponding integration anchors. Gene expression values were scaled and the top 30 principal components were retained to be used in downstream analysis.

#### Cell type annotation of snRNA-seq data

To visualize the transcriptomic landscape, a Uniform Manifold Approximation and Projection (UMAP) was used.^71^ First, a Batch Balance K-Nearest Neighbour graph (BBKNN) was generated using the aforementioned batch corrected principal components.^72^ Then, a UMAP layout was computed using scanpy_’s implementation with parameter ‘min_dist = 0.5’.

Leiden clusters were computed from the BBKNN graph with parameter ‘resolution = 1’, resulting in 25 clusters. Gene markers for each cluster were extracted with the function ‘findMarkers’ from scran_1.20.1 R package. These clusters were then manually annotated using a complementary approach based on gene set enrichment analysis with gprofiler,^53^ visualization of known markers and literature review. Cell cycle scores were calculated based on a predefined list of cell cycle genes associated with S and G2M phases. These scores were also used to refine cell type annotations (Table S3).

#### Cell type label transferring

Seurat_4.0.3 R package was used to transfer annotations from one dataset to another (i.e., from *in vivo* snRNA-seq experiments to organoids snRNA-seq, from the latter to single cell spatial transcriptomics, or from droplet snRNA-seq to split-seq dataset). Integrating anchors were obtained following the procedure for batch correction described above but instead of rpca, canonical correlation analysis (‘method=cca’) was used. The reference dataset was chosen depending on the annotations to be transferred.

#### Post-Analysis quality control

Differential expression analysis revealed a cluster of cells (Leiden cluster 12, Figures S3A and S3B) statistically enriched for biosynthesis and metabolic gene sets (high biosynthesis cluster). For instance, a large number of ribosomal proteins were detected. This specific cell set also showed sparse expression of gene markers present across all other known cell types, as well as notably low Seurat mapping scores of integration with *in vivo* snRNA-seq data from human neocortex (Figure S3C). These observations suggested the presence of either a spurious cell type arising from a technical artifact that was not detected in previous QC steps, or a heavily affected cell type likely exclusive of these organoids as has been previously suggested.^10^

It has been reported that droplet-based snRNA-seq is particularly subject to contamination by high amounts of ambient RNA.^73^ To discard this possibility, a simulation of highly contaminated cells was performed by summing up a fixed number of cell-wise randomly permuted count matrices (Figures S3D and S3E) and then downsampling the resulting counts by a fraction number equal to the number of matrices added (Figure S3E). Importantly, the cluster enriched for biosynthesis processes was left out. Nonetheless, when mapping the simulated data against the original dataset, cells displaying low library complexity and library size were consistently and largely associated with this cluster. Further downsampling showed that practically every cell can be allocated to this cluster when the dataset becomes too sparse (Figure S3E). Thus, because this cluster can be recovered by simulating contaminated cells taken from other clusters, it is with high probability a spurious cell type. Since in general highly and widely expressed genes are more likely to be contaminating, the expression profile of such an artifact is expected to include genes such as those associated with ribosomal proteins.

#### Differential abundance analysis

Differential abundance was tested using two alternative approaches, MiloR^31^ and scProportionTest.^32^ The former is cluster free and therefore independent of cell type annotations. The method creates a kNN graph to define neighbourhoods of interconnected cells. It then fits a general linear model to test for differential abundance between factors (i.e., treatment or morphology) assuming a negative binomial distribution for the cell abundances. It adjusts for multiple testing hypotheses by computing a spatial FDR value. The use of general linear models allows taking into account the variability between samples (e.g., Abundance = B0 + B1 treatment + B2 sample). The latter applies a permutation test over the annotation labels to compute a p-value and confidence intervals by bootstrapping. Here, 10,000 permutations were performed. This is a complementary approach, which helps in terms of interpretability.

#### Estimation of latent time using RNA velocity

RNA velocity as implemented in scVelo_0.2.4 was used to estimate gene-specific latent time-points obtained from the ‘dynamical’ model.^50^ These are then coupled to a universal gene-shared latent time, which represents the cell’s internal clock and is based only on its transcriptional dynamics, but that aims to resemble the course of cell differentiation. Root cells were by default selected based on a markov diffusion process.

#### Analysis of spatial transcriptomics data

We used RImageJROI for processing Molecular Cartography regions of interest (ROIs). ROIs (i.e., cells) with less than 11 mRNA molecules and less than 11 different genes detected were filtered out from the analysis. Moreover, cells from the spatial transcriptomics landscape mapping to the cluster identified by highly contaminated cells in snRNA-seq were excluded. Raw counts were then normalized using the function ‘logNormalize’ from scran_1.20.1 R package. If technical replicates were available, their spatial coordinates were aligned by their centroids and superimposed to be used for downstream analysis.

#### Projection of snRNA-seq onto spatial map

Numerical data such as RNA velocity latent time or gene expression levels were integrated into the spatial transcriptomics landscape using Seurat_4.0.3 R package in a similar way as the workflow described for label transferring, but instead of using directly log transformed library size normalized counts, the ‘scTransform’ algorithm was applied. Further, the top 30 principal components were computed and the values corresponding to k nearest neighbours (with k=30) in the snRNA-seq data were averaged and projected onto the spatial landscapes.

#### Spatial autocorrelation analysis

Spatial autocorrelation analysis was used to assess whether pre-selected genes selected for spatial transcriptomics provided sufficient information to describe the main anatomical structures of brain organoids as well as to identify other potential spatially variable markers. First, spatial neighbouring relationships between cells were stored in a Spatial Weighting Matrix (SWM) using the function ‘gabrielneigh’ from the spdep_x R package. Then, for each gene the Moran’s coefficient (MC) was computed to quantify the degree of dependency among cells in a spatial context and a randomization procedure was applied to compute a p-value in order to test for statistical significance. The function ‘moran.randtest’ from the spdep_x R package was used for this purpose. The resulting p-values were then adjusted for multiple testing hypotheses using a FDR approach. Following the same idea of latent time integration, projected gene expression values from snRNA-seq into spatial transcriptomics could be tested as spatially variable markers.

#### Spatially explicit dimensionality reduction

We used MULTISPATI (Multivariate spatial analysis based on Moran’s index) as implemented in the R package adespatial to perform spatially explicit dimensionality reduction on the spatial transcriptomics landscapes. Whereas the standard analysis such as PCA identifies the main structures, MULTISPATI seeks for spatial structures by extending MC to the multivariate case.^46^

#### *In vivo* transcriptional identity similarity

Morphological differences between individual organoids were summarized using PCA. The top two principal components were then used for k-means clustering (with K=2) revealing a split between samples associated with high and low morphology scores. These two clusters were used to perform differential abundance analysis between the organoid samples as well as to contrast the mapping quality for transferred annotations from *in vivo* datasets. Three *in vivo* datasets publicly available were selected for this task.^28^^,^^34^^,^^35^ Cell type annotations from these datasets were transferred to the organoid samples using Seurat as previously described. Then, a Wilcoxon rank sum test was used to assess whether the overall quality of cell type mapping (Seurat cell type mapping score) between high and low morphology score clusters was different. In all cases, a significant shift between the corresponding probability density functions was detected (p-value<0.01e-16) for which the high morphology cluster was consistently associated with better mapping quality. To compare the overall gene expression signatures from individual organoids with *in vivo* scRNA-seq data from Polioudakis et al.^28^, we integrated both datasets by normalising with scTransform function from the Seurat package and calculated the average gene expression across all cells for each sample (individual organoids and *in vivo* data). Human neocortex tissue gestational week (GW) 17-18 from Polioudakis et al.^28^ was profiled using scRNA-seq with Drop-seq/Fluidigm technology (33,976 cells). Prefrontal cortex (PFC) samples GW (5-37] from Nowakowski et al.^34^ was profiled using scRNA-seq with Fluidigm technology (4,262 cells). Human forebrain post-conceptional week (PCW) 8-10 from Van Bruggen et al.^35^ was profiled using scRNA-seq with 10X Genomics (25,161 cells).

#### GOterm analysis

Gene ontology analysis was performed for snRNA-seq cluster annotation, in parallel with manual annotation. For Figures S2E and S2F, the top 50 upregulated genes from cluster 2 (differentiating RG) and cluster 8 (high transcription RG) (Figure S3A) according to LogFC, FDR, p-value, were the input for gene ontology analysis on gProfiler.^74^ The top 5 GO:BP (biological process) were plotted on a bar plot graph. -Log(adjusted p-value) was displayed on the x axis. Anatomical structure morphogenesis (GO:0009653) was omitted from the bar plot for GO:BP enriched in differentiating RG as non-informative.

Gene ontology analysis was performed on differentially expressed (DE) genes upregulated in LSM versus HSM for cell clusters shown to be significantly enriched in LSM through Milo-test and scProportion test, namely RG, differentiating RG, IPCs, DL neurons, UL neurons. Top upregulated genes were selected through a cut-off LogFC=0.3. The genes were analysed through gProfiler. Resulting GOterms are listed in Tables S4A–S4D.

Gene ontology analysis was performed on top upregulated genes in mixed identity RG/Neu 1 versus cycling RG and pooled neuronal clusters respectively. Genes were selected through a cut-off LogFC=0.3 and analysed through gProfiler (Tables S6). Top 5 GO:BP (biological process) were plotted in bar plot graph (Figure 6B). -Log(adjusted p-value) was displayed on the x axis. GO:BP for nervous system development, anatomical structure morphogenesis, anatomical structure development were omitted as non-informative.

#### Latent time progression

Differential gene expression analysis was performed with findMarkers function from scran R package, to identify set genes associated with LSM and HSM. First, we split the snRNA-seq data into neuronal types and progenitors, leaving out Chp and cortical hem. We tested these subsets independently for differential expression between samples with LSM and HSM. Second, the expression values of these genes were smoothed in latent time order using windows of 1000 and scaled for further analysis. We computed the mean and standard deviation of pooled gene expression values to visualise the different trends of gene expression arising from differences in morphology.

#### Processing of Split-seq data

Sequence reads from single cell and single nuclei data were demultiplexed using demuxFQ into 8 and 2 sub-libraries respectively, allowing for one mismatch in the barcode sequences. Then, each sub-library was processed using the computational pipeline provided by Parse Biosciences, spipe v.1.0.3 and the GRCh38.108 genome reference. Initially, single cell barcodes with more than 400 genes, library sizes greater than 500 transcripts and a genes/library size ratio lower than 90% were retained. Similarly, single nuclei barcodes with more than 500 genes and library size greater than 1000 transcripts were retained. In both cases, cells with mitochondrial fraction greater than 20% were filtered out. Doublet calling was performed using scDblFinder^75^ with a doublet rate expectation of 5% for single cell and 3% for single nuclei. The latter is the expected rate reported by Parse Biosciences, however, due to difficulties during cell dissociation such expectation was higher for single cell data. Furthermore, we applied DecontX,^76^ a Bayesian method to remove RNA contamination in individual cells. To begin with, the fraction of contamination was calculated using decontX function and leiden clusters generated from highly variable genes as well as known cell type markers. To enforce a stringent decontamination procedure, delta, the concentration parameters for the Dirichlet prior to the contamination in each cell was fixed to 1 and 10 while the estimations obtained by default parameters were 1.23 and 0.99. After decontamination, only cells with more than 500 genes and library sizes greater than 700 transcripts were retained. The resulting number of barcodes was 14,198 for single cell and 14,767 for single nuclei, as for single nuclei there was no need for decontamination. Library size normalization was then performed using logNormCounts function from scran package. Due to the fixing of samples before library preparation with Parse Biosciences kits, all time points were processed and sequenced simultaneously. Hence, no batch correction was required. Cell type label transferring from 10x to Parse Biosciences data was performed using Seurat package as described above for 10x data sets, as is the case of differential abundance analysis with MiloR. DotPlot was computed using DotPlot function on Seurat_v4.3.0. Average expression and percent of cells expressing the selected genes identified by scRNA-seq were shown.

### Quantification and statistical analysis

All biological experiments were performed with multiple independent biological samples as detailed in the figure legends and STAR Methods section. Numbers of samples are detailed in figure legends for each quantification performed, including numbers of ventricles, organoids, and batches for each condition. Statistical test details can be found in the figure legends and STAR Methods. For comparisons across multiple samples, multiple comparisons corrections were applied as detailed in figure legends and STAR Methods. Statistics were performed and graphs were generated in Prism v9.5.1 (Graphpad) and using the above detailed packages in R.

## Data Availability

• Single-cell RNA-seq data have been deposited at the National Center for Biotechnology Information BioProjects Gene Expression Omnibus (GEO) under accession number GEO:GSE241543. Accession numbers are listed in the key resources table. • *In vivo* single-cell RNAseq data were from Polioudakis et al.,^28^ Nowakowski et al.,^34^ and Van Bruggen et al*.*^35^ • Microscopy data reported in this paper will be shared by the lead contact upon request. • All original RNA-seq code has been deposited at https://github.com/rosshandler/Structure-Indentity and is publicly available as of the date of publication. The DOI is listed in the key resources table. • All original Fiji/ImageJ code has been deposited at https://github.com/jboulanger/Organoid_morphology and is publicly available as of the date of publication. The DOI is listed in the key resources table. • Any additional information required to reanalyze the data reported in this paper is available from the lead contact upon request.

## Supplemental information

Document S1. Figures S1–S6 and Tables S1–S3 and S5

Table S4. Biological processes enriched in LSM overrepresented cell clusters, related to Figure 3 We selected clusters enriched in LSM organoids following the differential abundance analysis performed with MiloTest. We then looked at overexpressed genes in individual over-represented clusters of LSM organoids and performed GOterm analysis (GProfiler). The resultant GOterms are listed in the csv files (Tables S4A–S4D). 4A: RG cluster, 4B: differentiating RG cluster, 4C: DL neurons, 4D: UL neurons. RG=radial glia; DL=deep layer neurons; UL=upper layer neurons.

Table S6. Gene ontology analysis for upregulated genes in mixed identity cells, related to Figure 6 GOterm analysis (GProfiler) was performed on top upregulated genes in mixed identity RG/Neu cluster 1 versus cycling RG and pooled neuronal clusters respectively. Genes were selected through a cut-off LogFC=0.3. Genes were analysed through GProfiler and tables represent the top GOterms.

Methods S1. List of probes used for Molecular Cartography, related to STAR Methods Table containing the gene names, ENSEMBL IDs and Resolve Catalogue numbers for the Oligonucleotide probes used for gene targeting studies of Molecular Cartography, related to STAR Methods.

Document S2. Article plus supplemental information
