# Supplementary material for: Tissue morphology influences the temporal program of human brain organoid development
Source: Cell Stem Cell. Author manuscript; Available in PMC 2024 Jan 11. (PMC10765088; doi:10.1016/j.stem.2023.09.003)
Supplement: Document S1. Figures S1–S6 and Tables S1–S3 and S5 [file EMS192828-supplement-Document_S1__Figures_S1_S6_and_Tables_S1_S3_and_S5.pdf]

Cell Stem Cell, Volume *30*

Supplemental Information

Tissue morphology influences the temporal program of human brain organoid development

Ilaria Chiaradia, Ivan Imaz-Rosshandler, Benedikt S. Nilges, Jerome Boulanger, Laura Pellegrini, Richa Das, Nachiket D. Kashikar, and Madeline A. Lancaster

Supplementary Figure 1. Embryoid body patterning and late growth factor application influence organoid morphology

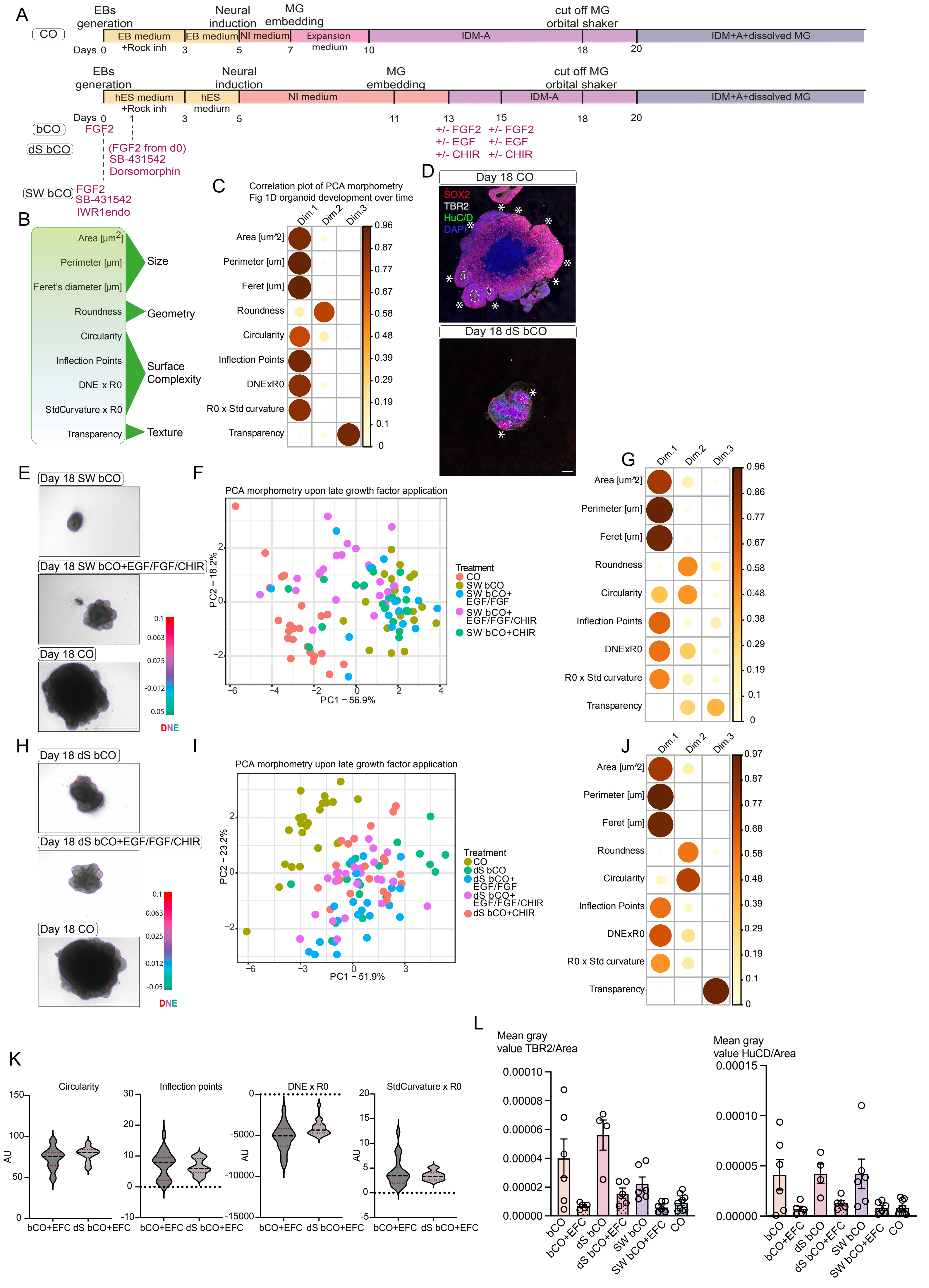

Supplementary Figure 2. Cell type annotation for snRNA-seq dataset

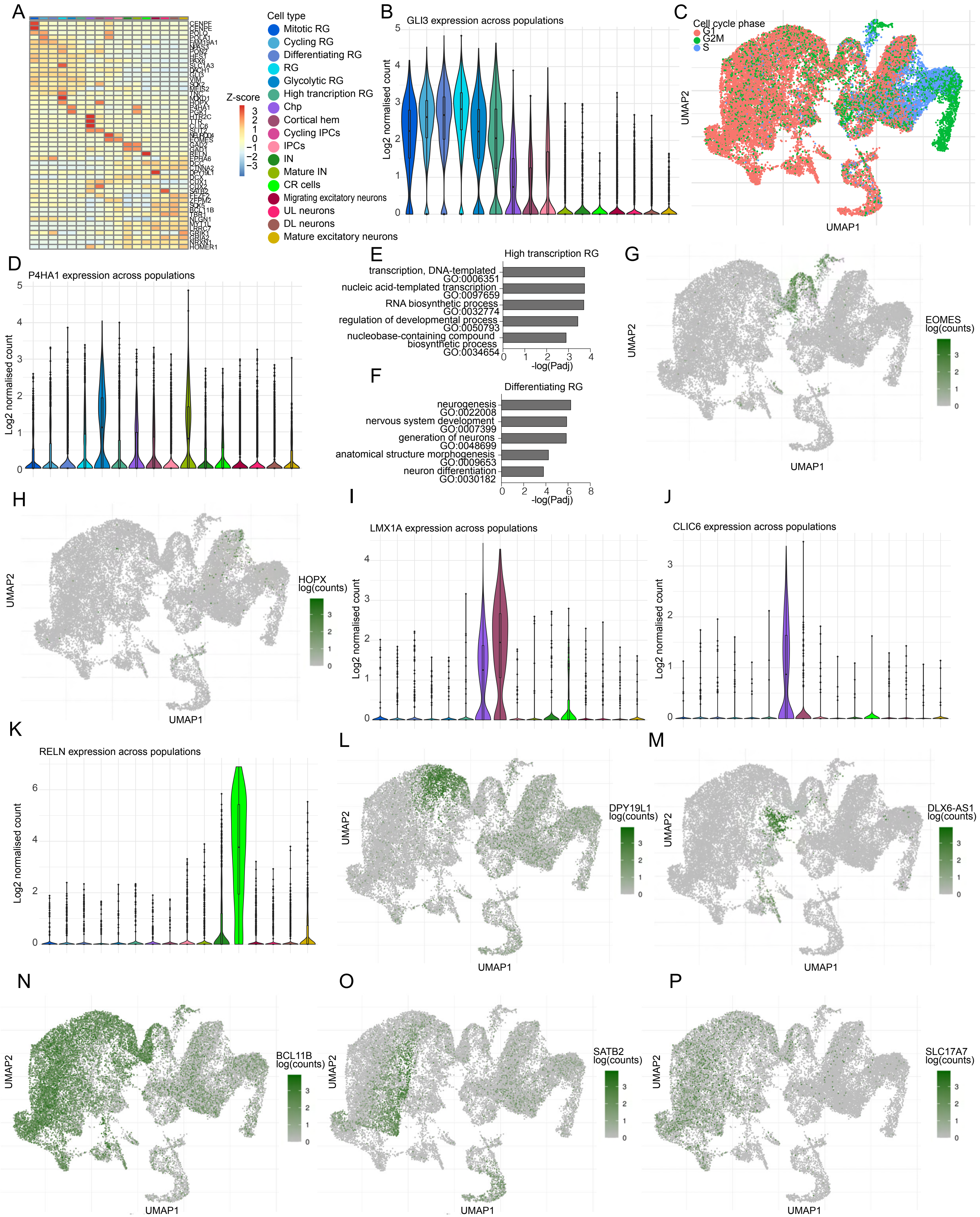

Supplementary Figure 3. Quality control of ambient RNA contamination

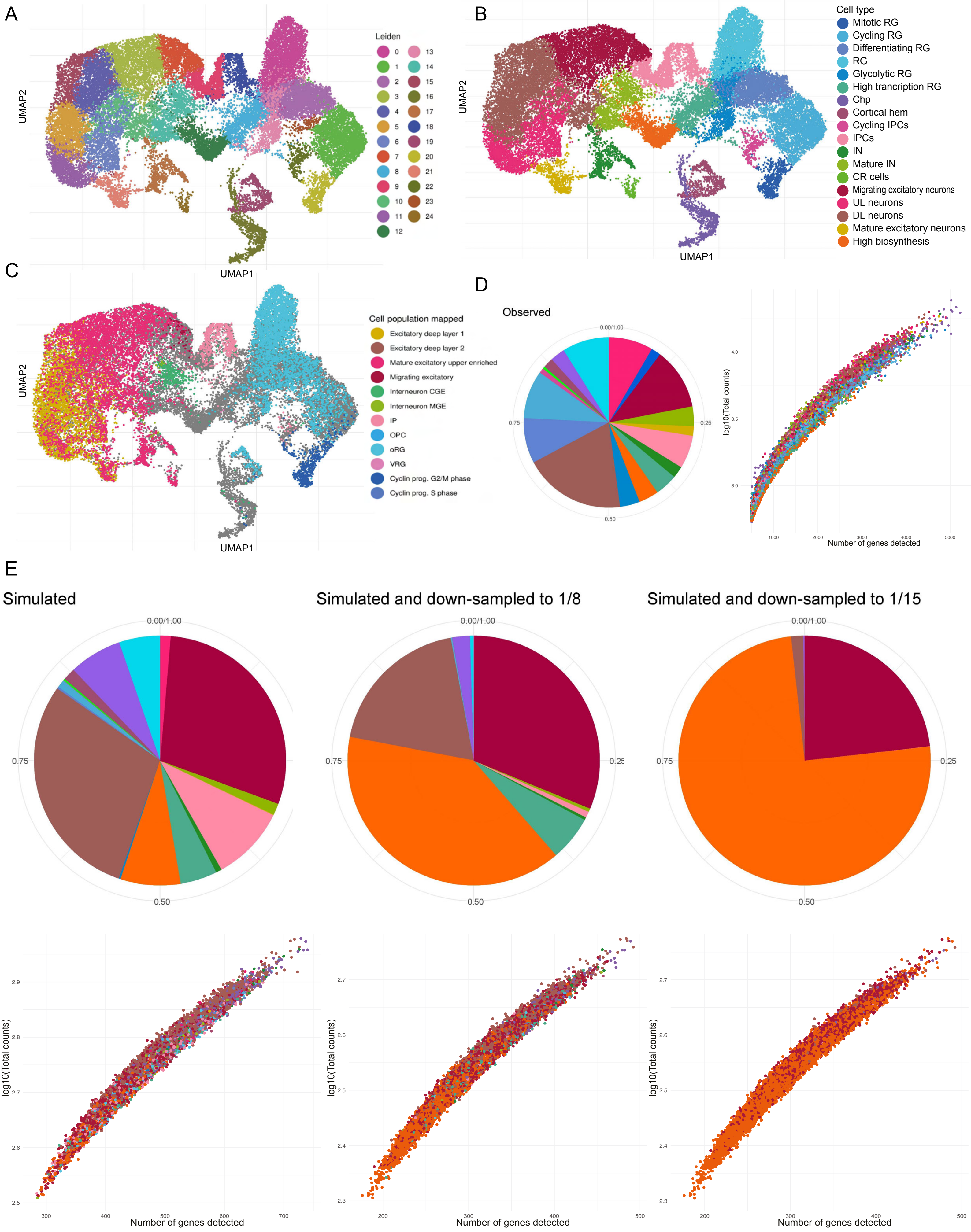

Supplementary Figure 4. Changes in organoid transcriptome linked to diversity in morphology

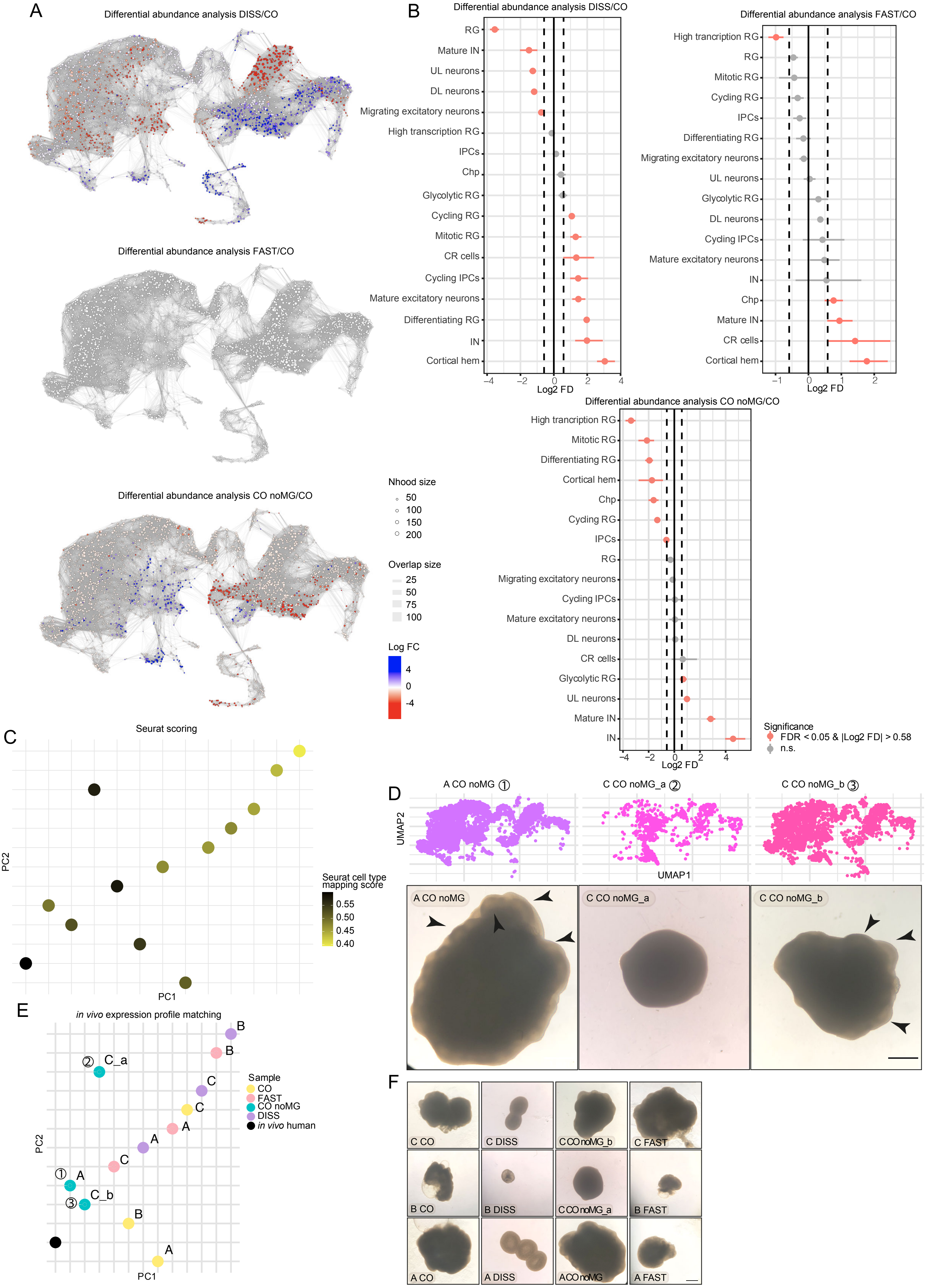

Supplementary Figure 5. Spatial transcriptomics of organoids with complex and simple cytoarchitecture

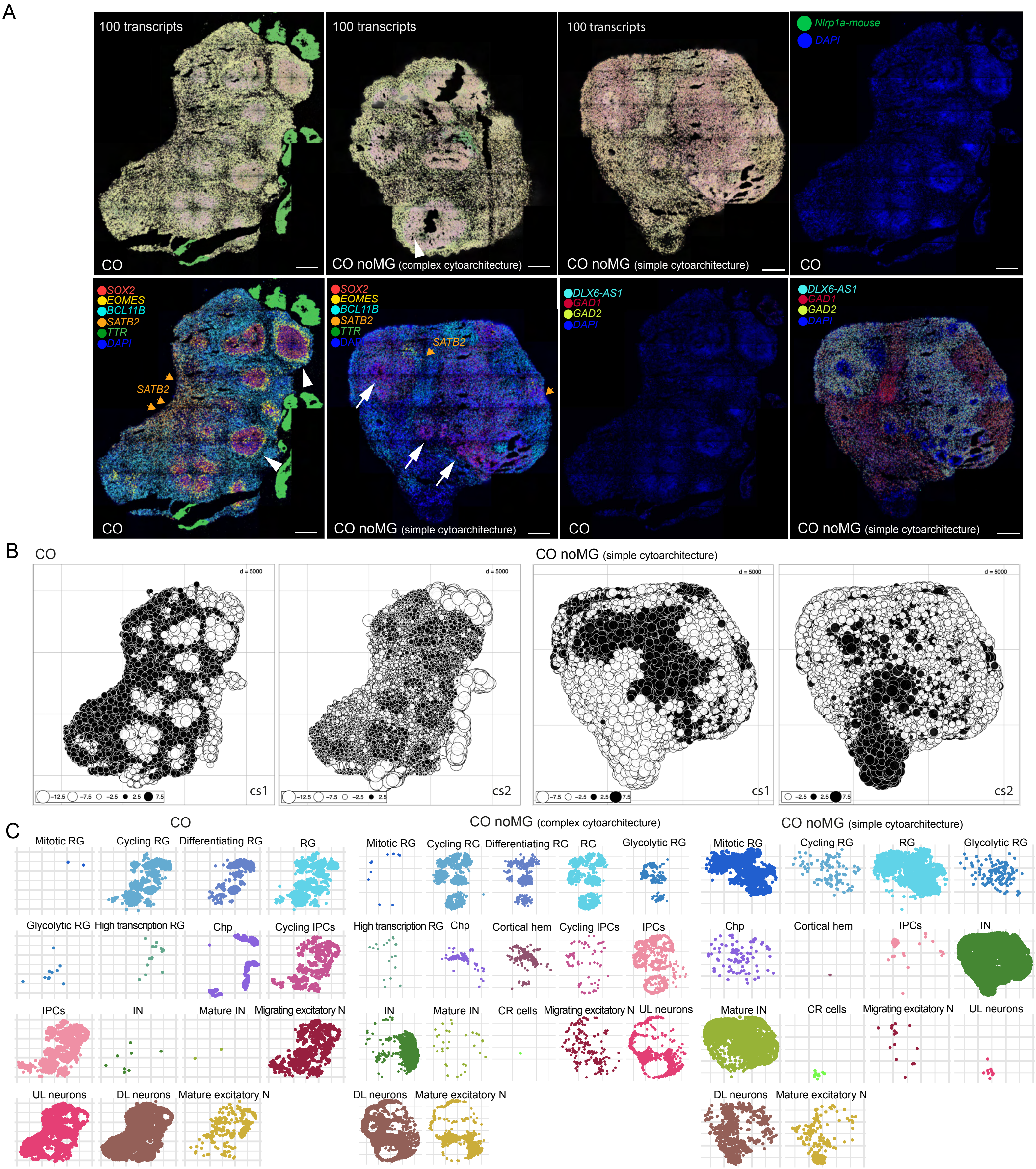

Supplementary Figure 6. Analysis of the developmental trajectory of LSM and HSM organoids

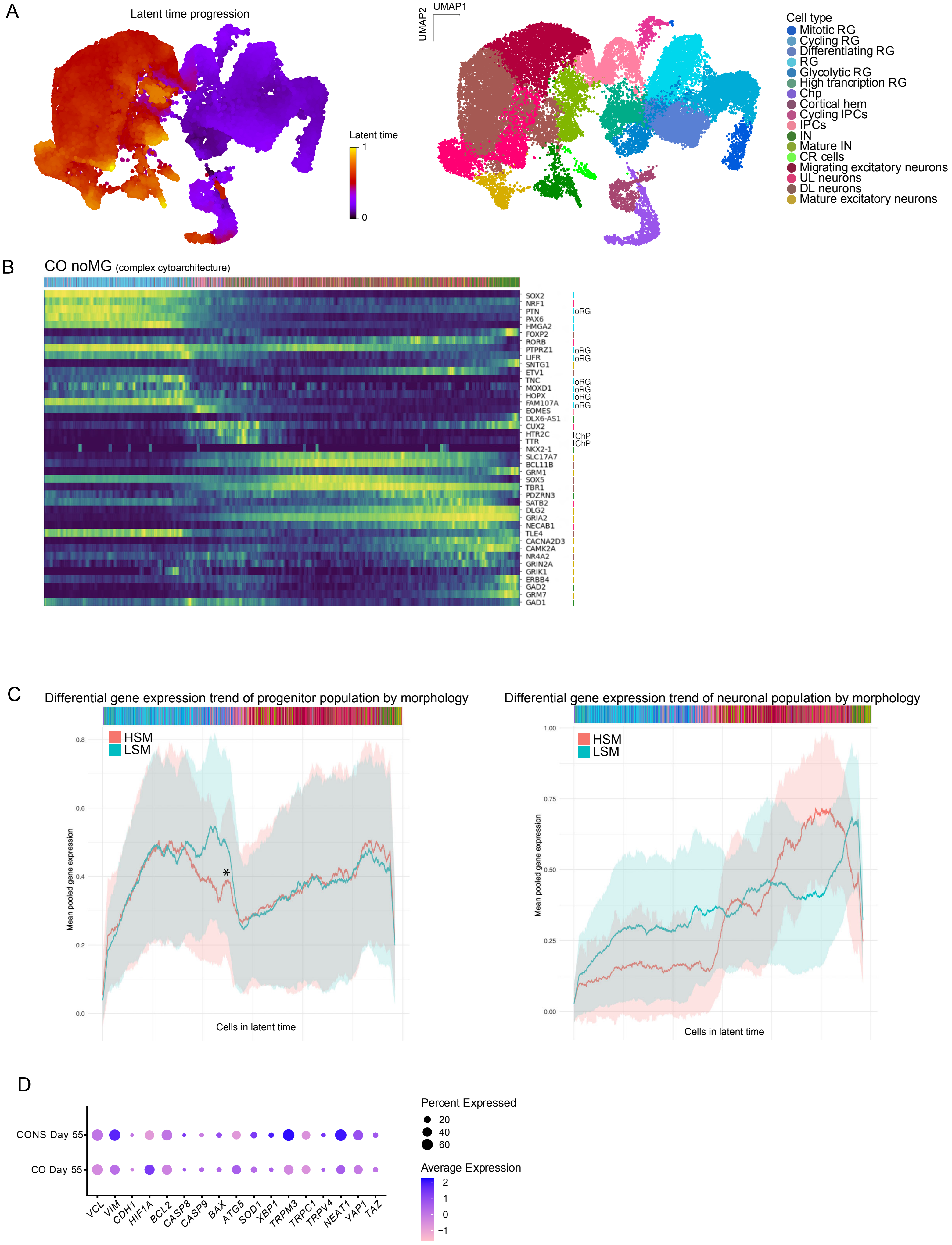

Supplementary Figure legends

Supplementary Figure 1. Embryoid body patterning and late growth factor application influence organoid morphology (related to Figure 1). A. Detailed schematics of organoid protocol and variables tested. CO: unguided protocol generated with STEMdiff™ Cerebral Organoid Kit. bCO: unguided protocol in basal media with low dose FGF2 as in *Lancaster et al., 2013* from day 0 to day 3. dS bCO: guided protocol in basal media (containing low dose FGF2 from day 0 to 3) with dualSMAD inhibition (SB-431542, Dorsomorphin) initiated at day 1 to day 5. SW bCO: guided protocol in basal media (containing low dose FGF2 from day 0 to 3) with TGFβ (SB-431542) and WNT (IWR1endo) inhibition from day 0 to day 5. When pulses of EGF/FGF2/CHIR were given, they were applied at day 13 and day 15. MG=Matrigel. **B.** List of the morphological features measured through the unbiased semi-automated morphometric pipeline. **C.** Correlation plot of the principle component analysis (PCA) of organoid development over time (Figure 1D) showing the contribution of each morphometric parameter to the PCA analysis as the squared cosine of each parameter across principal components. Colour intensity and size of the circles are proportional to the contribution of each parameter to the principal components. **D.** Immunohistochemistry of day 18 unguided CO, guided dS bCO showing progenitors (SOX2+), intermediate progenitors (TBR2+), neurons (HuC/D+), and nuclei staining DAPI. Neural tube-like units are shown by asterisks and ventricles outlined by dotted line. **E.** Representative brightfield images of SW bCO, SW bCO treated with EGF/FGF/CHIR, and CO at day 18 processed through the morphometric analysis. Gradient scale indicates DNE, red circles indicate Inflection Points. DNE=Dirichlet Normal Energy. **F.** PCA morphospace of day 18 CO, untreated SW bCO, and treated SW bCO (SW bCO+EGF/FGF, SW bCO+EGF/FGF/CHIR, or SW bCO+CHIR). Data points represent single organoids. Principal component (PC) axes display the percentage of variation. Six batches per condition were analysed. **G.** Correlation plot of the PCA of day 18 SW bCO untreated, SW bCO+EGF/FGF/CHIR, SW bCO+EGF/FGF, SW bCO+CHIR, and CO. **H.** Representative brightfield images of CO, dS bCO untreated, dS bCO+EGF/FGF/CHIR at day 18 processed through the morphometric analysis. Gradient scale indicates DNE, red circles indicate Inflection Points. **I.** PCA morphospace of day 18 CO, untreated dS bCO, and treated dS bCO (dS bCO+EGF/FGF/CHIR, dS bCO+EGF/FGF, dS bCO+CHIR). PC axes display the percentage of variation. Data points represent single organoids. Six batches per condition were analysed. **J.** Correlation plot of the PCA analysis of day 18 treated and untreated dS bCO. **K.** Violin plots of Circularity, Inflection Points, DNE x R0, StdCurvature x R0 for day 18 bCO treated with EGF/FGF/CHIR and dS bCO treated with EGF/FGF/CHIR. Median and quartiles are shown as dotted lines. Coefficient of variation Circularity: bCO+EFC 17.69%, dS bCO+EFC 10.97%; Coefficient of variation Inflection Points: bCO+EFC 61.41%, dS bCO+EFC 39.50%; Coefficient of variation DNE x R0: bCO+EFC 31.42%, dS bCO+EFC 22.81%; Coefficient of variation StdCurvature x R0: bCO+EFC 69.52%, dS bCO+EFC 26.84%; Six batches per condition were analysed. **L.** Quantitative analysis of TBR2 and HuC/D staining intensity. Mean gray value for these two markers was normalised on the organoid area. Data points represent single organoids from 3 batches per condition. Mean and standard error of the mean (SEM) are shown. Kruskal-Wallis test and Dunn's multiple comparisons test were performed. Scale bars: E., H. 1000μm; D. 100μm.

Supplementary Figure 2. Cell type annotation for snRNA-seq dataset (related to Figure 3). A. Heatmap of characteristic gene markers across cell populations, displaying genes in each row with Z-scaled normalized mean expression values. ChP = choroid plexus; IPCs = intermediate progenitor cells; IN = inhibitory neurons; UL = upper layer neurons; DL = deep layer neurons; CR = Cajal Retzsius. **B.** Violin plot with colour-coded cell types showing *GLI3* expression (radial glial [RG] marker) across cell clusters. **C.** Uniform Manifold Approximation and Projection (UMAP) plot with cells coloured by cell cycle phase. **D.** Violin plot of colour-coded clusters showing *P4HA1* expression (glycolytic RG marker) **E.** Gene ontology (GO) term analysis showing upregulated GO:BP (biological process) terms for high transcription RG cluster and the respective -log(adjusted *P*-value) (-log(Padj)). **F.** GO term analysis showing upregulated GO:BP terms for differentiating RG cluster and the respective -log(Padj). **G.** UMAP feature plot for *EOMES* (*TBR2*) expression (IPCs marker). **H.** UMAP feature plot for *HOPX* expression (outer radial glial marker). **I.** Violin plot colour-coded by cluster showing *LMX1A* expression (cortical hem marker). **J.** Violin plot colour-coded by cluster showing *CLIC6* expression (ChP marker). **K.** Violin plot colour-coded by cluster showing *RELN* expression (CR cells marker). **L.** UMAP feature plot for *DPY19L1* expression (migrating neurons marker). **M.** UMAP feature plot for *DLX6-AS1* expression (IN marker). **N.** UMAP feature plot for *BCL11B* expression (DL marker). **O.** UMAP feature plot for *SATB2* expression (UL marker). **P.** UMAP feature plot for *SLC17A7* expression (mature excitatory neurons marker).

Supplementary Figure 3. Quality control of ambient RNA contamination (related to Figure 3). A. Uniform Manifold Approximation and Projection (UMAP) plot of all unfiltered cells passing initial quality control, with cells coloured by Leiden clusters. **B.** UMAP plot of unfiltered cells coloured by cell type annotation, including Cluster 12, annotated as ‘High biosynthesis’. **C.** UMAP plot of unfiltered cells coloured by transferred cell type labels from *Polioudakis et al.*, 2019. Cells below a Seurat mapping score of 0.5 are shown in gray. Note the lack of mapping cells in Cluster 12. **D.** Pie plot displaying the proportion of cell type annotations (left). Library complexity shown as the number of genes per total UMI count in each cell (right). **E.** Proportion of cell type annotations and library complexity observed after simulating contaminated cells and progressive downsampling (from left to right). Despite leaving out Cluster 12 from our simulations (see methods), ‘High biosynthesis’ annotated cells are consistently and increasingly associated with low quality libraries, indicating these are likely ambient RNA contaminated droplets.

Supplementary Figure 4. Changes in organoid transcriptome linked to diversity in morphology (related to Figure 3). A. Differential abundance analysis of DISS versus CO, FAST versus CO (no statistically significant differences were observed), and CO noMG versus CO performed with MiloR on snRNA-seq data at day 55. The UMAP plot shows cell neighbourhood connections using a shared nearest neighbour graph, highlighting over-represented (in blue) and under-represented (in red) regions in the transcriptomic landscape of DISS organoids. Nodes are neighbourhoods (Nhood), coloured by their log fold change across conditions. Non-Differentially Abundant (DA) neighbourhoods (FDR 5%) are coloured in white, and sizes correspond to the number of cells in a neighbourhood. Graph edges depict the number of cells shared between adjacent neighbourhoods. **B.** Permutation-based (scProportionTest) cell type composition analysis of DISS versus CO, FAST versus CO, and CO noMG versus CO. Significantly enriched populations are highlighted in

red. FD=Fold difference; FDR=false discovery rate. **C.** PCA plot as in Figure 3C with samples coloured by the median Seurat cell type mapping score against the *in vivo* landscape. The value for *in vivo* was set to 0.6, which here represents the optimal score. The *in vivo* human developing brain scRNA-seq dataset plotted is *Polioudakis et al*., *2019*. **D.** UMAP plot and brightfield pictures of individual day 55 CO noMG samples linking transcriptional profile to morphology. Arrowheads indicate neural tube-like units. **E.** PCA plot as in Figure 3C of mean gene expression values where single CO noMG data points are highlighted with number labels 1-3 corresponding to number labels 1-3 in panel D. The *in vivo* human developing brain scRNA-seq dataset plotted is *Polioudakis et al*., *2019*. Note how organoids 1 and 3 cluster closer to the *in vivo* data point compared to organoid 2. **F.** Brightfield images of day 55 organoids used for snRNA-seq and morphometric analysis in Figure 3 and assessed for their similarity to *in vivo* transcriptional landscape.

Supplementary Figure 5. Spatial transcriptomics of organoids with complex and simple cytoarchitecture (related to Figure 4). A. Spatial distribution of 100 selected transcripts in CO and CO noMG (Molecular Cartography). From left to right, top to bottom: all 100 transcripts; *Nlrp1a*-mouse specific negative control; *SOX2/EOMES/BCL11B/SATB2/TTR*; inhibitory neuron markers *DLX6-AS1/GAD1/GAD2*. Orange arrows indicate *SATB2*+ cells in CO and CO noMG. White arrowheads show examples of large ventricles, white arrows point to neural rosette-like units **B.** Spatially explicit dimensionality reduction performed with MULTISPATI applied to CO and CO noMG highlighting the structures captured by the two top dimensions (cs1, cs2). The dot size is proportional to MULTISPATI scores (analogous to PCA scores) and highlights spatial patterns associated with gene expression. **C.** Spatial transcriptomics landscape split by cell type cluster across different samples highlights differences in cell type abundance and structural composition. The different number of panels reflects the differential representation of specific cells types. Scalebar: A. 200µm.

Supplementary Figure 6. Analysis of the developmental trajectory of LSM and HSM organoids (related to Figure 4, 5). A. Uniform Manifold Approximation and Projection (UMAP) plot with cells coloured by universal gene-shared latent time modelling the course of cell differentiation (left panel). UMAP plot by cell type clusters (right panel). RG=radial glia; IPCs=intermediate progenitor cells; DL= deep layer neurons; UL=upper layer neurons; IN=inhibitory neurons; ChP= choroid plexus; CR=Cajal-Retzius cells. **B.** Heatmap displaying the expression of highly variable genes from the spatial transcriptomic landscape of CO noMG with complex cytoarchitecture with cells ordered by latent time. Individual genes are highlighted as markers of specific cell clusters shown by colour code. The top colour bar shows each cell colored by cell cluster assignment. oRG=outer radial glia. **C.** Gene expression trend of upregulated genes in LSM and HSM organoids identified within progenitor population (left panel) and within neuronal population (right panel). Cells are ordered by latent time. The trends are computed as the mean across pooled genes from the differential expression analysis. Asterisk labels IPC stage. **D.** Dot plot of constrained (CONS) and CO day 55 showing average expression and percent of cells expressing a set of candidate genes previously implicated in force transmission and cell stress.

Supplementary Table 1. Key variables in neural organoid protocols (related to Figure 1A)

	Variable	Reference
EBs patterning	DMEM-F12	[13,14,15,24,27]
	Glasgow-MEM	[8,25]
	KOSR	[8,13,15,24,25,27]
	FBS	[13,15]
	Y-27632	[8,13,14,15,25,27]
	FGF2	[13,15,24]
	TGFβ inhibitor	[8,14,24,25,27]
	BMP inhibitor	[14,24,27]
	WNT inhibitor	[8,25,27]
EBs preparation	Single cell suspension	[8,13,15,25,27]
	Colonies	[14,24]
Neural induction	Neurobasal	[14]
	DMEM-F12	[8,13,15,24,25]
	B-27-A supplement	[14,27]
	B27+A supplement	[24]
	N2 supplement	[8,13,15,24,25,27]
	TGFβ inhibitor	[24]
	WNT activators	[24]
	Heparin	[13,15,24]
	Insulin	[24,27]
	FGF2	[14]
	EGF	[14]
	KOSR	[13]
	FBS	[13]
Neuroepithelium expansion	Embedding in MG	[13,15,24]
	Dissolved MG	[25]
	WNT agonist	[16]

Supplementary Table 2. Conditions tested for their role in influencing organoid morphology early on and in mature organoids (related to Figure 1A, 2G)

Condition	Experiment	EB stage	NE expansion stage	Morphological perturbation	References
Basal cerebral organoid (bCO)	Common protocol variations affect organoid morphology (Figure 1)	hESC medium + ROCK inhibitor Y27632 50µM + bFGF 4ng/mL (day 0 to 3). From day 3 Y27632 and bFGF were removed	MG embedding<br><br>IDM-A (bCO)<br><br>+EGF/+FGF/+CHIR (bCO+EFC)<br><br>+EGF/+FGF (bCO+EF)<br><br>+CHIR (bCO+C)<br><br>Pulses given at day 13 and 15	NA	[15]
dualSMAD inhibition organoid (dS bCO)	Common protocol variations affect organoid morphology (Figure 1)	hESC medium + ROCK inhibitor Y27632 50µM + bFGF 4ng/mL. From day 1 to 5 10µM SB 431542 + 1µM Dorsomorphin. From day 3 Y27632 and bFGF were removed	MG embedding<br><br>IDM-A (dS bCO)<br><br>+EGF/+FGF/+CHIR (dS bCO+EFC)<br><br>+EGF/+FGF (dS bCO+EF)<br><br>+CHIR (dS bCO+C)<br><br>Pulses given at day 13 and 15	NA	[14,15,24,25,27]
SMAD WNT inhibition organoid (SW bCO)	Common protocol variations affect organoid morphology (Figure 1)	hESC medium + ROCK inhibitor Y27632 50µM + bFGF 4ng/mL). From day 0 to 5 10µM SB 431542 + 3µM IWR1endo. From day 3 Y27632 and bFGF were removed	MG embedding<br>IDM-A (SW bCO)<br>+EGF/+FGF/+CHIR (SW bCO+EFC)<br>+EGF/+FGF (SW bCO+EF)<br>+CHIR (SW bCO+C)<br>Pulses given at day 13 and 15	NA	[8,15,25,27]
Control cerebral organoid (CO)	Common protocol variations affect organoid morphology (Figure 1)	STEMdiff™ Cerebral Organoid Kit, StemCell Technologies 08570. ROCK inhibitor Y27632 50µM from day 0 to day 3	MG embedding<br><br>STEMdiff™ Cerebral Organoid Kit, StemCell Technologies 08570	NA	[15]
Control cerebral	Variables influencing morphology in	STEMdiff™ Cerebral Organoid Kit,	MG embedding	MG embedding	[15]

organoid (CO)	mature organoids (Figure 2)	StemCell Technologies 08570. ROCK inhibitor Y27632 50µM from day 0 to day 3	STEMdiff™ Cerebral Organoid Kit, StemCell Technologies 08570	STEMdiff™ Cerebral Organoid Kit, StemCell Technologies 08570<br><br>IDM+A+MG Orbital shaker speed 57 rpm (Φ25 cm)	
Control cerebral organoid noMG (CO noMG)	Variables influencing morphology in mature organoids (Figure 2)	STEMdiff™ Cerebral Organoid Kit, StemCell Technologies 08570. ROCK inhibitor Y27632 50µM from day 0 to day 3	No MG embedding<br><br>STEMdiff™ Cerebral Organoid Kit, StemCell Technologies 08570	No MG embedding<br><br>STEMdiff™ Cerebral Organoid Kit, StemCell Technologies 08570<br><br>IDM+A Orbital shaker speed 57 rpm (Φ 25 cm)	[14,15]
Control cerebral organoid fast shaking speed (FAST)	Variables influencing morphology in mature organoids (Figure 2)	STEMdiff™ Cerebral Organoid Kit, StemCell Technologies 08570. ROCK inhibitor Y27632 50µM from day 0 to day 3	MG embedding<br><br>STEMdiff™ Cerebral Organoid Kit, StemCell Technologies 08570	MG embedding<br><br>STEMdiff™ Cerebral Organoid Kit, StemCell Technologies 08570<br><br>IDM+A+MG Orbital shaker speed 78 rpm (Φ 25 cm)	[10]

Supplementary Table 3. Cell cycle related genes (related to Supplementary Figure 3B)
Cell cycle phase specific genes guided UMAP cluster annotation according to the cell cycle phase. Cell cycle scores were calculated based on a predefined list of cell cycle genes associated with S and G2M phases.

**Phase_S**	**Phase_G2M**
MCM5	CDK1
PCNA	NUSAP1
TYMS	UBE2C
FEN1	BIRC5
MCM2	TPX2
MCM4	TOP2A
RRM1	NDC80
UNG	CKS2
GINS2	NUF2
MCM6	CKS1B
CDCA7	MKI67
DTL	TMPO
PRIM1	CENPF
UHRF1	TACC3
MLF1IP	FAM64A
HELLS	SMC4
RFC2	CCNB2
RPA2	CKAP2L
NASP	CKAP2
RAD51AP1	AURKB
GMNN	BUB1
WDR76	KIF11
SLBP	ANP32E
CCNE2	TUBB4B
UBR7	GTSE1
POLD3	KIF20B
MSH2	HJURP
ATAD2	CDCA3
RAD51	HN1
RRM2	CDC20
CDC45	TTK
CDC6	CDC25C
EXO1	KIF2C
TIPIN	RANGAP1
DSCC1	NCAPD2
BLM	DLGAP5
CASP8AP2	CDCA2

USP1	CDCA8
CLSPN	ECT2
POLA1	KIF23
CHAF1B	HMMR
BRIP1	AURKA
E2F8	PSRC1
HMGB2	ANLN
	LBR
	CKAP5
	CENPE
	CTCF
	NEK2
	G2E3
	GAS2L3
	CBX5
	CENPA

Supplementary Table 5. Moran's Coefficient applied to spatial transcriptomics (related to extended data figure 5B)

For each of the selected genes used for Molecular Cartography, we computed the Moran's coefficient (MC) to quantify the degree of dependency among cells in a spatial context. A randomization procedure was applied to compute a *P*-value in order to test for statistical significance. The function 'moran.randtest' from the spdep_R package was used for this purpose. The resulting *P*-values were then adjusted for multiple testing hypotheses using a FDR approach. The higher the MC, the more representative genes are for the spatial landscape.

Gene	MC	Alter	Pvalue	FDR
**ADAMTS19**	0.052138281	greater	0.001	0.001265823
**ADGRL3**	0.25184882	greater	0.001	0.001265823
**ALDH1L1**	0.015803424	greater	0.002	0.0025
**AQP4**	-0.00327581	greater	0.741	0.763917526
**AUTS2**	0.61136223	greater	0.001	0.001265823
**BCL11B**	0.483834831	greater	0.001	0.001265823
**CACNA2D3**	0.122998894	greater	0.001	0.001265823
**CAMK2A**	0.05223383	greater	0.001	0.001265823
**CLDN11**	0.004164808	greater	0.157	0.178409091
**CUX1**	0.250328077	greater	0.001	0.001265823
**CUX2**	0.253300849	greater	0.001	0.001265823
**DLG2**	0.402885133	greater	0.001	0.001265823
**DLG4**	0.384784668	greater	0.001	0.001265823
**DLX6-AS1**	-0.00083894	greater	0.382	0.410752688
**EOMES**	0.415960692	greater	0.001	0.001265823
**ERBB4**	0.127084712	greater	0.001	0.001265823
**ETV1**	0.144590704	greater	0.001	0.001265823
**FAM107A**	0.352778482	greater	0.001	0.001265823
**FEZF2**	0.260479745	greater	0.001	0.001265823
**FOXA2**	-0.000314717	greater	0.514	0.541052632
**FOXG1**	0.280504523	greater	0.001	0.001265823
**FOXP2**	0.142862536	greater	0.001	0.001265823
**FZD9**	0.005932506	greater	0.057	0.065517241
**GAD1**	0.139159252	greater	0.001	0.001265823
**GAD2**	0.013906289	greater	0.028	0.032941176
**GFAP**	0.051138386	greater	0.001	0.001265823
**GLI3**	0.647945023	greater	0.001	0.001265823
**GLRA3**	0.019768025	greater	0.001	0.001265823
**GORASP2**	0.051436902	greater	0.001	0.001265823
**GPHN**	0.034399495	greater	0.001	0.001265823
**GRIA2**	0.429930536	greater	0.001	0.001265823
**GRIK1**	-0.001004402	greater	0.965	0.974
**GRIN1**	0.334690214	greater	0.001	0.001265823

**GRIN2A**	0.066464109	greater	0.001	0.001265823
**GRIN2B**	0.396154514	greater	0.001	0.001265823
**GRM1**	0.049241415	greater	0.001	0.001265823
**GRM7**	0.081194989	greater	0.001	0.001265823
**HMGA2**	0.528260398	greater	0.001	0.001265823
**HOMER1**	0.132411337	greater	0.001	0.001265823
**HOPX**	0.116706062	greater	0.001	0.001265823
**HTR2C**	0.844957829	greater	0.001	0.001265823
**IL1RAPL2**	0.035658124	greater	0.001	0.001265823
**IL6ST**	0.16359811	greater	0.001	0.001265823
**ISL1**	0.040025721	greater	0.001	0.001265823
**LAMP5**	0.038447804	greater	0.001	0.001265823
**LEF1**	0.517607355	greater	0.001	0.001265823
**LIFR**	0.315994428	greater	0.001	0.001265823
**LIMS2**	0.010996785	greater	0.02	0.023809524
**MBP**	-0.000408902	greater	0.365	0.401098901
**MOXD1**	0.196809417	greater	0.001	0.001265823
**NECAB1**	0.119777863	greater	0.001	0.001265823
**NKX2-1**	-0.001119642	greater	0.507	0.539361702
**Nlrp1a-mouse**	-0.000118954	greater	0.916	0.934693878
**NR4A2**	0.209109132	greater	0.001	0.001265823
**NRF1**	0.110295018	greater	0.001	0.001265823
**NRG1**	0.123891507	greater	0.001	0.001265823
**NRP2**	0.211901397	greater	0.001	0.001265823
**NRXN3**	0.058263099	greater	0.001	0.001265823
**OLIG1**	0.005655897	greater	0.029	0.03372093
**OLIG2**	0.013691954	greater	0.019	0.022891566
**OTX1**	0.441991029	greater	0.001	0.001265823
**PAX2**	-0.000356777	greater	0.974	0.974
**PAX6**	0.511427571	greater	0.001	0.001265823
**PBX3**	0.078986489	greater	0.001	0.001265823
**PCDH15**	0.012386734	greater	0.005	0.00617284
**PDZRN3**	0.065791949	greater	0.001	0.001265823
**PGK 1.00**	0.02047768	greater	0.001	0.001265823
**PROX1**	0.037555098	greater	0.001	0.001265823
**PTN**	0.522963488	greater	0.001	0.001265823
**PTPRZ1**	0.400704356	greater	0.001	0.001265823
**PVALB**	0.016194324	greater	0.011	0.013414634
**RAB3B**	0.196560629	greater	0.001	0.001265823
**RELN**	0.111209813	greater	0.001	0.001265823
**RORB**	0.042939253	greater	0.001	0.001265823
**RPL13**	0.039913644	greater	0.001	0.001265823
**RPL13A**	0.038015861	greater	0.001	0.001265823

**RPL7**	0.016401631	greater	0.001	0.001265823
**RPS19**	0.04906598	greater	0.001	0.001265823
**S100B**	0.034348044	greater	0.001	0.001265823
**SATB2**	0.554136762	greater	0.001	0.001265823
**SEMA3C**	0.201283923	greater	0.001	0.001265823
**SHANK3**	0.055251926	greater	0.001	0.001265823
**SIX3**	0.354443136	greater	0.001	0.001265823
**SLC17A7**	0.39312274	greater	0.001	0.001265823
**SLC6A3**	-0.000187058	greater	0.324	0.36
**SLC6A4**	0.000211316	greater	0.376	0.408695652
**SNAP25**	0.162991931	greater	0.001	0.001265823
**SNTG1**	0.031067185	greater	0.001	0.001265823
**SORCS2**	0.097639283	greater	0.001	0.001265823
**SOX2**	0.571551292	greater	0.001	0.001265823
**SOX5**	0.607144932	greater	0.001	0.001265823
**SST**	-0.000713386	greater	0.263	0.295505618
**SYP**	0.249830086	greater	0.001	0.001265823
**TBR1**	0.634978106	greater	0.001	0.001265823
**TH**	0.105318353	greater	0.001	0.001265823
**TLE4**	0.159942514	greater	0.001	0.001265823
**TNC**	0.135008756	greater	0.001	0.001265823
**TPH2**	-0.00013532	greater	0.583	0.607291667
**TTR**	0.920920553	greater	0.001	0.001265823
**UNC5C**	0.019116264	greater	0.001	0.001265823
